# A dataset on void ratio limits and their range for cohesionless soils

**DOI:** 10.1016/j.dib.2019.104696

**Published:** 2019-10-25

**Authors:** Makbule Ilgac, Gizem Can, Kemal Onder Cetin

**Affiliations:** Dept. of Civil Engineering, Middle East Technical University, Ankara, Turkey

**Keywords:** Void ratio, Mean grain size, Coefficient of uniformity

## Abstract

A database, which consists of maximum and minimum void ratio limits and their range, particle size, distribution and shape characteristics, is compiled. More specifically, minimum and maximum void ratios (e_min_ and e_max_) along with their range (e_max_-e_min_), particle roundness (R) and spherecity (S), fines content (FC), coefficient of uniformity (C_u_), mean grain size (D_50_) data are compiled from natural cohesionless soils and reconstituted grained material (e.g.: rice, glass beads, mica) mixtures. The final dataset is composed of 636, mostly soil samples. Out of 636 samples, 496, 474 and 603 of them have e_max_, e_min_ or e_max_-e_min_ data, respectively. Similarly, for 593, 419, 171, 126 and 93 soils, D_50_, C_u_, R, S and FC data exists, respectively. Not for every sample, USCS based soil classification designation is available, hence for the missing ones, soil classification is performed based on mean particle diameter-based classification as suggested by ASTM D2487 – 17: Standard Practice for Classification of Soils for Engineering Purposes (Unified Soil Classification System) [1]. The dataset consists of 19 silts and clays, 527 sands (357 fine sands, 153 medium sands, 17 coarse sands) and 47 gravels (44 fine gravels, 3 coarse gravels). A spreadsheet summary of the dataset is provided. This dataset is later used for the development of probability-based void ratio predictive models.

**Table:** Specifications Table

Subject	• *Earth and Planetary Sciences* • *Engineering*
Specific subject area	• *Geotechnical Engineering and Engineering Geology* • *Civil and Structural Engineering*
Type of data	*Table*
How data were acquired	*The index parameters presented in this article were compiled from available literature in the form of articles, engineering reports, etc., where void ratio limits and their range along with their grain size, distribution and shape characteristics of cohesionless soils are determined by laboratory tests. These laboratory tests are referred to as standard test methods for particle-size distribution (gradation) of soils using sieve analysis, standard test methods for laboratory determination of density (unit weight) of soil specimens etc.*
Data format	*Raw, filtered*
Parameters for data collection	*The parameters of the database are selected as minimum and maximum void ratio (e*_max_*, e*_min_*) along with their range (e*_*max*_*-e*_*min*_*), mean grain size D*_*50*_*, uniformity coefficient C*_*u*_*, fines content FC, roundness R, and spherecity S.*
Description of data collection	*The database is compiled from existing literature, which presents void ratio limits, grain size, distribution and shape characteristics determined by laboratory tests. Void ratio limits are estimated by mostly American Standards for Testing and Materials (ASTM) and Japanese Geotechnical Society standards (JGS). More specifically, followings are the test procedures used:* i) *standard test methods for particle-size distribution (gradation) of soils using sieve analysis,* ii *standard test methods for laboratory determination of density (unit weight) of soil specimens and* iii) *x-ray diffraction testing* *The classification of soil specimens is made based on Unified Soil Classification System (USCS) if available.*
Data source location	*Japan, USA, Turkey, Australia, Norway, Switzerland.*
Data accessibility	*Data is included in this article* *Supplementary A*
Related research article

**Table:** 

**Value of the Data** • Due to widely known difficulties in “undisturbed” sampling of cohesionless soils, their in-situ “density” states are determined by using their loosest and the densest void ratio limits along with their relative density state. • This dataset provides insights, which can be used by researchers and civil/geotechnical engineers to understand void ratio response governed by stress-state, grain size, distribution and shape parameters. • The dataset helps estimating in-situ void ratio and void ratio range of cohesionless soils through which stress-strain, and volumetric compression potential of them can be better understood. • The compiled dataset incorporates a large number of data points along with broad range of soil types including silt, fine-coarse sand and fine-coarse gravel. • Compared to currently available datasets, the major contributions of this study are that the dataset provides an extended-, well-documented and digitized (electronically available) data and the classification of the soil types based on ASTM D2487 – 17 [1]. • This dataset can be used for the development of new probabilistic and deterministic void ratio predictive relationships.

## 1 Data

This paper provides details about database compilation efforts including but not limited to index properties and void ratio limits of different soil types (clay, silt, sand, gravel). The data consists of a summary spreadsheet, where original and cross-references, soil type, soil classification and index properties: roundness (R), spherecity (S), fines content (FC), mean grain size distribution (D_50_), coefficient of uniformity (C_u_), minimum, maximum and range of void ratio (e_max_, e_min_, e_max_-e_min_) are presented. The final dataset is composed of 19 silts and clays, 527 sands (357 fine sands, 153 medium sands, 17 coarse sands) and 47 gravels (44 fine gravels, 3 coarse gravels) samples. When USCS-based soil classification designation is missing, samples are classified based on mean particle diameter relationship as suggested by ASTM D2487 – 17: Standard Practice for Classification of Soils for Engineering Purposes (Unified Soil Classification System) [1]. Table 1 summarizes simple statistics of database variables.

**Table 1:** A summary of database simple statistics.

	C_u_	D_50_ (mm)	FC %	R	S	e_min_	e_max_	e_max_-e_min_
**Number of Data**	419	593	93	171	126	474	496	603
**Mean (μ)**	7.38	1.30	10.10	0.44	0.73	0.55	0.92	0.39
**Median (M)**	2.00	0.34	2.50	0.41	0.72	0.55	0.91	0.36
**Standard deviation (σ)**	26.86	3.18	17.75	0.20	0.13	0.15	0.26	0.16
**Range (R)**	1.1–325.3	0.0–25.0	0.0–100.0	0.1–1.0	0.2–1.0	0.2–2.2	0.4–3.0	0.2–1.5

## 2 Experimental design, materials and methods

The compiled dataset presents a summary of the experimental findings including minimum and maximum void ratios (e_max_, e_min_) along with their range (e_max_-e_min_), grain size (represented by mean grain size D_50_), grain distribution (represented by coefficient of uniformity C_u_ and fines content, FC), and grain shape (represented by roundness R and spherecity S) parameters for different types of mostly cohesionless soils. The final dataset is composed of 60.2% fine sand, 25.8% medium sand, 2.9% coarse sand, 7.4% fine gravel and 0.5% coarse gravel data. The dataset is filtered and analyzed by using the Microsoft Excel software. This data article provides documentation of data for the development of new void ratio predictive models.

A summary of the data is given in Supplementary A. The output table in each spreadsheet contains the following columns:

• Data number
• Soil Type (Monterey no.0/30 Sand, Cambria Sand, Ottawa Sand etc.)
• Soil Classification (Fine Gravel, Coarse Sand etc.)
• Roundness, R
• Spherecity, S
• Fines content, FC
• Mean grain size distribution, D_50_ (mm)
• Coefficient of uniformity, C_u_
• Maximum void ratio, e_max_
• Minimum void ratio, e_min_
• Void ratio range, e_max_-e_min_
• Test Method (ASTM, JGS etc.)
• Reference
