# Supplementary material for: A dataset on void ratio limits and their range for cohesionless soils
Source: Data Brief. 2019 Oct 25;27:104696. doi: 10.1016/j.dib.2019.104696 (PMC6859216; doi:10.1016/j.dib.2019.104696)
Supplement: Multimedia component 1 [file mmc1.docx]

| ***Data #*** | ***Soil Type*** | ***Soil Classification*** | ***R*** | ***S*** | ***FC*** | ***D_50_ (mm)*** | ***Cu*** | ***e_max_*** | ***e_min_*** | ***e_max_-e_min_*** | ***Test Methodology*** | ***Reference*** |
| --- | --- | --- | --- | --- | --- | --- | --- | --- | --- | --- | --- | --- |
| 1 | Silty Soils (30<FC<70%) |  |  |  |  | 0.042 |  |  |  | 0.83 | Japanese Geotecnical Society-Procedures | Cubrinovski and Ishiara (2002) |
| 2 | Silty Soils (30<FC<70%) |  |  |  |  | 0.044 |  |  |  | 0.77 | Japanese Geotecnical Society-Procedures | Cubrinovski and Ishiara (2002) |
| 3 | Silty Soils (30<FC<70%) |  |  |  |  | 0.049 |  |  |  | 0.92 | Japanese Geotecnical Society-Procedures | Cubrinovski and Ishiara (2002) |
| 4 | Nevada Fines | Silt |  |  | 100 | 0.050 | 1.667 | 1.18 | 0.75 | 0.42 | Non-Standard Procedures | Cubrinovski and Ishiara (2002), Lade et al. (1998) |
| 5 | Faro Lead-Zinc |  |  |  | 65 | 0.050 |  | 2.02 | 0.84 | 1.18 | NA | Jefferies and Been (2006), Golder Project Files |
| 6 | Sudbury (nickel) |  |  |  | 65 | 0.050 |  |  |  |  | NA | Jefferies and Been (2006), Golder Project Files |
| 7 | Silty Soils (30<FC<70%) |  |  |  |  | 0.054 |  |  |  | 0.87 | Japanese Geotecnical Society-Procedures | Cubrinovski and Ishiara (2002) |
| 8 | Merriespruit Gold Tailings |  |  |  | 60 | 0.060 |  | 1.83 | 0.66 | 1.17 | NA | Jefferies and Been (2006), Fourie & Papageorgiou (2001) |
| 9 | Silty Soils (30<FC<70%) |  |  |  |  | 0.063 |  |  |  | 0.81 | Japanese Geotecnical Society-Procedures | Cubrinovski and Ishiara (2002) |
| 10 | Silty Soils (30<FC<70%) |  |  |  |  | 0.064 |  |  |  | 0.83 | Japanese Geotecnical Society-Procedures | Cubrinovski and Ishiara (2002) |
| 11 | Silty Soils (30<FC<70%) |  |  |  |  | 0.068 |  |  |  | 0.72 | Japanese Geotecnical Society-Procedures | Cubrinovski and Ishiara (2002) |
| 12 | Silty Soils (30<FC<70%) |  |  |  |  | 0.071 |  |  |  | 0.92 | Japanese Geotecnical Society-Procedures | Cubrinovski and Ishiara (2002) |
| 13 | Silty Soils (30<FC<70%) |  |  |  |  | 0.075 |  |  |  | 0.68 | Japanese Geotecnical Society-Procedures | Cubrinovski and Ishiara (2002) |
| 14 | San Fernando 7 |  |  |  | 50 | 0.075 |  |  |  |  | NA | Jefferies and Been (2006), Seed et al. (1988) |
| 15 | Silty Soils (30<FC<70%) |  |  |  |  | 0.077 |  |  |  | 0.91 | Japanese Geotecnical Society-Procedures | Cubrinovski and Ishiara (2002) |
| 16 | Amauligak I-65 |  |  |  | 48 | 0.080 |  |  |  |  | NA | Jefferies and Been (2006), Golder Project Files |
| 17 | Silty Soils (30<FC<70%) |  |  |  |  | 0.083 |  |  |  | 0.80 | Japanese Geotecnical Society-Procedures | Cubrinovski and Ishiara (2002) |
| 18 | Silty Soils (30<FC<70%) |  |  |  |  | 0.085 |  |  |  | 0.65 | Japanese Geotecnical Society-Procedures | Cubrinovski and Ishiara (2002) |
| 19 | Granite Powder |  |  |  | 42 | 0.089 | 6.180 | 1.30 | 0.48 | 0.81 | NA | Santamarina and Cho (2001), Santamarina and Cho (2001) |
| 20 | Granite Powder |  | 0.4 | 0.24 |  | 0.090 | 6.200 | 1.30 |  |  | ASTM C136, D4254, and D1557 | Zheng and Hryciw (2016), Cho et al. (2006) |
| 21 | Silty Soils (30<FC<70%) |  |  |  |  | 0.091 |  |  |  | 0.62 | Japanese Geotecnical Society-Procedures | Cubrinovski and Ishiara (2002) |
| 22 | Silty Soils (30<FC<70%) |  |  |  |  | 0.095 |  |  |  | 0.87 | Japanese Geotecnical Society-Procedures | Cubrinovski and Ishiara (2002) |
| 23 | Del Monte white Sand |  |  |  |  | 0.096 | 1.400 | 1.20 | 0.63 | 0.58 | ASTM D 2049-69 | Youd (1973) |
| 24 | Crushed Basalt |  |  |  |  | 0.097 | 1.400 | 1.42 | 0.80 | 0.62 | ASTM D 2049-69 | Youd (1973) |
| 25 | Faro Lead-Zinc |  |  |  | 30 | 0.100 |  | 0.99 | 0.56 | 0.43 | NA | Jefferies and Been (2006), Golder Project Files |
| 26 | Silty Soils (30<FC<70%) |  |  |  |  | 0.100 |  |  |  | 0.74 | Japanese Geotecnical Society-Procedures | Cubrinovski and Ishiara (2002) |
| 27 | Silty Soils (30<FC<70%) |  |  |  |  | 0.100 |  |  |  | 0.69 | Japanese Geotecnical Society-Procedures | Cubrinovski and Ishiara (2002) |
| 28 | Yatesville Silty Sand |  |  |  | 43 | 0.100 |  |  |  |  | NA | Jefferies and Been (2006), Brandon et al. (1991) |
| 29 | Silty Soils (30<FC<70%) |  |  |  |  | 0.110 |  |  |  | 0.63 | Japanese Geotecnical Society-Procedures | Cubrinovski and Ishiara (2002) |
| ***Data #*** | ***Soil Type*** | ***Soil Classification*** | ***R*** | ***S*** | ***FC*** | ***D_50_ (mm)*** | ***Cu*** | ***e_max_*** | ***e_min_*** | ***e_max_-e_min_*** | ***Test Methodology*** | ***Reference*** |
| 30 | Merriespruit Gold Tailings |  |  |  | 30 | 0.110 |  | 1.33 | 0.58 | 0.75 | NA | Jefferies and Been (2006), Fourie & Papageorgiou (2001) |
| 31 | Sudbury (nickel) |  |  |  | 35 | 0.115 |  | 1.03 | 0.54 | 0.50 | NA | Jefferies and Been (2006), Golder Project Files |
| 32 | Silty Soils (30<FC<70%) |  |  |  |  | 0.116 |  |  |  | 0.92 | Japanese Geotecnical Society-Procedures | Cubrinovski and Ishiara (2002) |
| 33 | Silty Soils (30<FC<70%) |  |  |  |  | 0.118 |  |  |  | 0.60 | Japanese Geotecnical Society-Procedures | Cubrinovski and Ishiara (2002) |
| 34 | Ottawa F-110 Sand |  |  |  | 5 | 0.120 | 1.620 | 0.85 | 0.54 | 0.31 | NA | Santamarina and Cho (2001) |
| 35 | Ottawa F-110 Sand |  | 0.7 | 0.7 |  | 0.120 | 1.700 | 0.85 | 0.54 | 0.31 | ASTM C136, D4254, and D1557 | Zheng and Hryciw (2016), Cho et al. (2006) |
| 36 | Ottawa 100–140 |  | 0.5 | 0.9 |  | 0.120 | 1.200 | 0.92 | 0.54 | 0.38 | NA | Zheng and Hryciw (2016), Zelasko et al. (1975) |
| 37 | Nevada Sand 80/200 | SP |  |  | 0 | 0.120 | 1.625 | 0.94 | 0.62 | 0.32 | Non-Standard Procedures | Cubrinovski and Ishiara (2002), Lade et al. (1998) |
| 38 | Leighton Buzzard |  |  |  | 5 | 0.120 |  | 1.02 | 0.67 | 0.36 | NA | Jefferies and Been (2006), Golder Project Files |
| 39 | Sands with Clay (15<FC<30 %) |  |  |  |  | 0.123 |  |  |  | 0.57 | Japanese Geotecnical Society-Procedures | Cubrinovski and Ishiara (2002) |
| 40 | Silty Soils (30<FC<70%) |  |  |  |  | 0.127 |  |  |  | 0.59 | Japanese Geotecnical Society-Procedures | Cubrinovski and Ishiara (2002) |
| 41 | Silty Soils (30<FC<70%) |  |  |  |  | 0.129 |  |  |  | 0.71 | Japanese Geotecnical Society-Procedures | Cubrinovski and Ishiara (2002) |
| 42 | Ottawa 100–200 |  | 0.6 | 0.6 |  | 0.130 | 1.900 | 0.90 | 0.59 | 0.31 | NA | Zheng and Hryciw (2016), Thomann (1990) |
| 43 | Jamuna Sand |  | 0.1 | 0.68 |  | 0.130 | 1.900 | 1.14 | 0.72 | 0.42 | NA | Zheng and Hryciw (2016), Yasin and Safiullah (2003) |
| 44 | Merriespruit Gold Tailings |  |  |  | 20 | 0.130 |  | 1.33 | 0.70 | 0.63 | NA | Jefferies and Been (2006), Fourie & Papageorgiou (2001) |
| 45 | Silty Soils (30<FC<70%) |  |  |  |  | 0.134 |  |  |  | 0.67 | Japanese Geotecnical Society-Procedures | Cubrinovski and Ishiara (2002), Cubrinovski and Ishiara (2002) |
| 46 | Alaskan Beaufort |  |  |  | 10 | 0.140 |  | 0.84 | 0.53 | 0.31 | NA | Jefferies and Been (2006), Golder Project Files |
| 47 | Alaskan Beaufort |  |  |  | 5 | 0.140 |  | 0.86 | 0.57 | 0.29 | NA | Jefferies and Been (2006), Golder Project Files |
| 48 | Merriespruit Gold Tailings |  |  |  | 0 | 0.140 |  | 1.22 | 0.74 | 0.48 | NA | Jefferies and Been (2006), Fourie & Papageorgiou (2001) |
| 49 | Amauligak F-24 |  |  |  | 10 | 0.140 |  |  |  |  | NA | Jefferies and Been (2006), Golder Project Files |
| 50 | Sands with Clay (15<FC<30 %) |  |  |  |  | 0.141 |  |  |  | 0.62 | Japanese Geotecnical Society-Procedures | Cubrinovski and Ishiara (2002) |
| 51 | Amauligak F-24 |  |  |  | 21 | 0.144 |  |  |  |  | NA | Jefferies and Been (2006), Golder Project Files |
| 52 | Clean Sands (FC=0-5%) |  |  |  |  | 0.149 |  |  |  | 0.43 | Japanese Geotecnical Society-Procedures | Cubrinovski and Ishiara (2002) |
| 53 | Castro Sand B |  |  |  | 0 | 0.150 |  | 0.84 | 0.50 | 0.34 | NA | Jefferies and Been (2006), Castro (1969) |
| 54 | Nevada Sand |  | 0.6 | 0.85 |  | 0.150 | 1.800 | 0.85 | 0.57 | 0.28 | ASTM C136, D4254, and D1557 | Zheng and Hryciw (2016), Cho et al (2006) |
| 55 | P2-S7 |  | 0.22 |  |  | 0.150 | 3.200 | 0.86 | 0.52 | 0.34 | ASTM D 4254 and ASTM D 4253 | Zheng and Hryciw (2016), Bareither et al. (2008) |
| 56 | Nevada |  |  |  | 7.5 | 0.150 |  | 0.89 | 0.51 | 0.38 | NA | Jefferies and Been (2006), Velacs Project |
| 57 | Toyoura Sand | SP |  |  |  | 0.150 |  | 0.99 | 0.61 | 0.37 | Japanese Geotecnical Society-Procedures | Cubrinovski and Ishiara (2002), Shamoto et al. (1996) |
| 58 | Longstone Sand |  | 0.3 | 0.65 |  | 0.150 | 1.300 | 1.00 | 0.61 | 0.39 | Procedure proposed by Kolbuszewski (1948) | Zheng and Hryciw (2016), Tsomokos and Georgiannou (2010) |
| 59 | TKS Sand | SP |  |  |  | 0.150 | 1.880 | 1.02 | 0.60 | 0.43 | ASTM | Kokusho and Yoshida (1997) |
| 60 | Sands with Fines (5<FC<15%) |  |  |  |  | 0.152 |  |  |  | 0.51 | Japanese Geotecnical Society-Procedures | Cubrinovski and Ishiara (2002) |
| ***Data #*** | ***Soil Type*** | ***Soil Classification*** | ***R*** | ***S*** | ***FC*** | ***D_50_ (mm)*** | ***Cu*** | ***e_max_*** | ***e_min_*** | ***e_max_-e_min_*** | ***Test Methodology*** | ***Reference*** |
| 61 | Sands with Clay (15<FC<30 %) |  |  |  |  | 0.155 |  |  |  | 0.69 | Japanese Geotecnical Society-Procedures | Cubrinovski and Ishiara (2002) |
| 62 | Sands with Clay (15<FC<30 %) |  |  |  |  | 0.158 |  |  |  | 0.67 | Japanese Geotecnical Society-Procedures | Cubrinovski and Ishiara (2002) |
| 63 | Sands with Clay (15<FC<30 %) |  |  |  |  | 0.159 |  |  |  | 0.54 | Japanese Geotecnical Society-Procedures | Cubrinovski and Ishiara (2002) |
| 64 | Nevada Sand |  | 0.61 | 0.72 |  | 0.160 | 1.300 | 0.88 | 0.58 | 0.30 | NA | Zheng and Hryciw (2016) |
| 65 | Syncrude (Mildred Lake) |  |  |  | 10 | 0.160 |  | 0.96 | 0.52 | 0.44 | NA | Jefferies and Been (2006), Robertson et al. (2000) |
| 66 | P2-S3 |  | 0.24 |  |  | 0.160 | 2.300 | 0.96 | 0.58 | 0.38 | ASTM D 4254 and ASTM D 4253 | Zheng and Hryciw (2016), Bareither et al. (2008) |
| 67 | Toyoura Sand |  | 0.35 | 0.65 |  | 0.160 | 1.500 | 0.97 | 0.61 | 0.36 | NA | Zheng and Hryciw (2016), Bolton (1987) |
| 68 | Toyoura Sand | SP |  |  |  | 0.160 | 1.460 | 0.97 | 0.61 | 0.36 | Japanese Geotecnical Society-Procedures | Cubrinovski and Ishiara (2002), Hoque and Tatsuoka (1998) |
| 69 | Toyoura Sand | SP |  |  |  | 0.160 | 1.460 | 0.98 | 0.61 | 0.37 | Japanese Geotecnical Society-Procedures | Cubrinovski and Ishiara (2002), Goto et al. (1993) |
| 70 | Toyoura Sand | SP |  |  |  | 0.160 |  | 0.98 | 0.61 | 0.37 | Japanese Geotecnical Society-Procedures | Cubrinovski and Ishiara (2002), Sakai and Tanaka (1998) |
| 71 | Toyoura Sand |  | 0.3 |  |  | 0.160 | 1.500 | 0.98 | 0.61 | 0.37 | NA | Zheng and Hryciw (2016), Herle and Gudehus (1999) |
| 72 | Toyoura |  |  |  | 0 | 0.160 |  | 0.98 | 0.61 | 0.37 | NA | Jefferies and Been (2006), Golder Project Files |
| 73 | Sands with Fines (5<FC<15%) |  |  |  |  | 0.161 |  |  |  | 0.47 | Japanese Geotecnical Society-Procedures | Cubrinovski and Ishiara (2002) |
| 74 | Sands with Fines (5<FC<15%) |  |  |  |  | 0.161 |  |  |  | 0.44 | Japanese Geotecnical Society-Procedures | Cubrinovski and Ishiara (2002) |
| 75 | Ottawa Sand F-95 | SP |  |  | 0 | 0.163 | 1.818 | 0.87 | 0.58 | 0.29 | Non-Standard Procedures | Cubrinovski and Ishiara (2002), Lade and Yamamuro (1997) |
| 76 | Sands with Fines (5<FC<15%) |  |  |  |  | 0.164 |  |  |  | 0.58 | Japanese Geotecnical Society-Procedures | Cubrinovski and Ishiara (2002) |
| 77 | Sands with Clay (15<FC<30 %) |  |  |  |  | 0.166 |  |  |  | 0.55 | Japanese Geotecnical Society-Procedures | Cubrinovski and Ishiara (2002) |
| 78 | Clean Sands (FC=0-5%) |  |  |  |  | 0.167 |  |  |  | 0.43 | Japanese Geotecnical Society-Procedures | Cubrinovski and Ishiara (2002) |
| 79 | Ooestershelde |  |  |  |  | 0.170 | 1.800 | 0.89 | 0.56 | 0.33 | NA | Mayne and Kulhawy (1991), Greeuw et al. (1988) |
| 80 | S. Oakleigh Fine |  |  |  |  | 0.170 | 1.600 | 0.93 | 0.57 | 0.36 | NA | Mayne and Kulhawy (1991), Veismanis (1974) |
| 81 | Toyoura Sand | SP |  |  |  | 0.170 |  | 0.98 | 0.60 | 0.38 | Japanese Geotecnical Society-Procedures | Cubrinovski and Ishiara (2002), Gutierrez et al. (1991) |
| 82 | Toyoura Sand | SP |  |  | 0 | 0.170 | 1.583 | 0.99 | 0.62 | 0.37 | Japanese Geotecnical Society-Procedures | Cubrinovski and Ishiara (2002), Zlatovic (1994) |
| 83 | Jekyll Island Sand |  | 0.3 | 0.85 |  | 0.170 | 1.700 | 1.04 |  |  | ASTM C136, D4254, and D1557 | Zheng and Hryciw (2016), Cho et al. (2006) |
| 84 | Sands with Clay (15<FC<30 %) |  |  |  |  | 0.179 |  |  |  | 0.60 | Japanese Geotecnical Society-Procedures | Cubrinovski and Ishiara (2002) |
| 85 | Silty Soils (30<FC<70%) |  |  |  |  | 0.179 |  |  |  | 0.69 | Japanese Geotecnical Society-Procedures | Cubrinovski and Ishiara (2002) |
| 86 | Ottawa 70–100 |  | 0.5 | 0.9 |  | 0.180 | 1.200 | 0.92 | 0.54 | 0.38 | NA | Zheng and Hryciw (2016), Zelasko et al. (1975) |
| 87 | Evanston Beach 70–100 |  | 0.42 | 0.72 |  | 0.180 | 1.200 | 0.93 | 0.53 | 0.40 | NA | Zheng and Hryciw (2016), Zelasko et al. (1975) |
| 88 | Toyoura Sand | SP |  |  |  | 0.180 | 1.500 | 0.97 | 0.62 | 0.35 | Japanese Geotecnical Society-Procedures | Cubrinovski and Ishiara (2002), Yamashita and Toki (1993) |
| 89 | Toyoura Sand | SP |  |  |  | 0.180 | 1.200 | 0.97 | 0.64 | 0.34 | Japanese Geotecnical Society-Procedures | Cubrinovski and Ishiara (2002), Hyodo et al. (1991) |
| ***Data #*** | ***Soil Type*** | ***Soil Classification*** | ***R*** | ***S*** | ***FC*** | ***D_50_ (mm)*** | ***Cu*** | ***e_max_*** | ***e_min_*** | ***e_max_-e_min_*** | ***Test Methodology*** | ***Reference*** |
| 90 | Toyoura Sand | SP |  |  |  | 0.180 |  | 0.97 | 0.64 | 0.34 | Japanese Geotecnical Society-Procedures | Cubrinovski and Ishiara (2002), Zhang et al. (1997) |
| 91 | Toyoura Sand | SP |  |  |  | 0.180 | 1.300 | 0.98 | 0.61 | 0.37 | Japanese Geotecnical Society-Procedures | Cubrinovski and Ishiara (2002), Miura and Kawamura (1996) |
| 92 | Toyoura Sand | SP |  |  |  | 0.180 | 1.790 | 0.99 | 0.62 | 0.37 | Japanese Geotecnical Society-Procedures | Cubrinovski and Ishiara (2002), Zen and Yamazaki (1990) |
| 93 | Ponte Vedra Sand |  | 0.3 | 0.85 |  | 0.180 | 1.800 | 1.07 |  |  | ASTM C136, D4254, and D1557 | Zheng and Hryciw (2016), Cho et al. (2006) |
| 94 | Syncrude Tailings |  | 0.2 | 0.62 |  | 0.180 | 2.500 | 1.14 | 0.59 | 0.55 | NA | Zheng and Hryciw (2016), Sukumaran and Ashmawy (2001) |
| 95 | Niigata LD3-S2 |  |  |  |  | 0.180 | 1.800 | 1.26 | 0.66 | 0.60 | Japanese Standards | Ishiara et al (1978), Ishiara et al (1978) |
| 96 | Sands with Clay (15<FC<30 %) |  |  |  |  | 0.182 |  |  |  | 0.63 | Japanese Geotecnical Society-Procedures | Cubrinovski and Ishiara (2002) |
| 97 | Clean Sands (FC=0-5%) |  |  |  |  | 0.183 |  |  |  | 0.43 | Japanese Geotecnical Society-Procedures | Cubrinovski and Ishiara (2002) |
| 98 | Sands with Clay (15<FC<30 %) |  |  |  |  | 0.184 |  |  |  | 0.56 | Japanese Geotecnical Society-Procedures | Cubrinovski and Ishiara (2002) |
| 99 | Ottowa Sand | SP |  |  |  | 0.185 | 1.400 | 0.82 | 0.45 | 0.36 | ASTM D 2049-69 | Youd (1973) |
| 100 | Crushed Basalt |  |  |  |  | 0.185 | 1.400 | 1.34 | 0.74 | 0.60 | ASTM D 2049-69 | Youd (1973) |
| 101 | Del Monte white Sand |  |  |  |  | 0.187 | 1.400 | 1.07 | 0.55 | 0.53 | ASTM D 2049-69 | Youd (1973) |
| 102 | Clean Sands (FC=0-5%) |  |  |  |  | 0.190 |  |  |  | 0.46 | Japanese Geotecnical Society-Procedures | Cubrinovski and Ishiara (2002) |
| 103 | Clean Sands (FC=0-5%) |  |  |  |  | 0.190 |  |  |  | 0.38 | Japanese Geotecnical Society-Procedures | Cubrinovski and Ishiara (2002) |
| 104 | Sands with Fines (5<FC<15%) |  |  |  |  | 0.190 |  |  |  | 0.50 | Japanese Geotecnical Society-Procedures | Cubrinovski and Ishiara (2002) |
| 105 | Mol Sand |  | 0.65 | 0.65 |  | 0.190 | 1.500 | 0.89 | 0.56 | 0.33 | NA | Zheng and Hryciw (2016), DeBeer (1963) |
| 106 | Toyoura Sand | SP |  |  |  | 0.190 | 1.700 | 0.99 | 0.62 | 0.37 | Japanese Geotecnical Society-Procedures | Cubrinovski and Ishiara (2002), Cubrinovski and Ishiara (1998) |
| 107 | Clean Sands (FC=0-5%) |  |  |  |  | 0.193 |  |  |  | 0.36 | Japanese Geotecnical Society-Procedures | Cubrinovski and Ishiara (2002) |
| 108 | P2-S10 |  | 0.31 |  |  | 0.200 | 2.300 | 0.75 | 0.46 | 0.29 | ASTM D 4254 and ASTM D 4253 | Zheng and Hryciw (2016), Bareither et al. (2008) |
| 109 | P2-S2 |  | 0.29 |  |  | 0.200 | 2.100 | 0.83 | 0.56 | 0.27 | ASTM D 4254 and ASTM D 4253 | Zheng and Hryciw (2016), Bareither et al. (2008) |
| 110 | Sizewell |  |  |  |  | 0.200 | 2.000 | 0.94 | 0.50 | 0.44 | Japanese Society for Soil Mechanics and Foundation Engineering, 1979 | Skempton (1986) |
| 111 | Hochstetten Sand |  | 0.3 |  |  | 0.200 | 1.600 | 0.95 | 0.55 | 0.40 | NA | Zheng and Hryciw (2016), Herle and Gudehus (1999) |
| 112 | Hilton Mines |  | 0.23 | 0.72 | 2.5 | 0.200 | 2.000 | 1.05 | 0.62 | 0.43 | NA | Mayne and Kulhawy (1991), Schmertmann (1978) |
| 113 | Highland Valley Copper |  |  |  | 8 | 0.200 |  | 1.06 | 0.54 | 0.51 | NA | Jefferies and Been (2006), Robertson et al. (2000) |
| 114 | Fraser River |  |  |  | <5 | 0.200 |  | 1.10 | 0.70 | 0.40 | NA | Jefferies and Been (2006), Robertson et al. (2000) |
| 115 | Duncan Dam |  |  |  | 6.5 | 0.200 |  | 1.15 | 0.76 | 0.39 | NA | Jefferies and Been (2006), Robertson et al. (2000) |
| 116 | Sands with Clay (15<FC<30 %) |  |  |  |  | 0.200 |  |  |  | 0.59 | Japanese Geotecnical Society-Procedures | Cubrinovski and Ishiara (2002) |
| ***Data #*** | ***Soil Type*** | ***Soil Classification*** | ***R*** | ***S*** | ***FC*** | ***D_50_ (mm)*** | ***Cu*** | ***e_max_*** | ***e_min_*** | ***e_max_-e_min_*** | ***Test Methodology*** | ***Reference*** |
| 117 | Ottawa Sand 50/200 | SP |  |  | 0 | 0.202 | 1.889 | 0.81 | 0.55 | 0.26 | Non-Standard Procedures | Cubrinovski and Ishiara (2002), Lade and Yamamuro (1997) |
| 118 | Sands with Fines (5<FC<15%) |  |  |  |  | 0.204 |  |  |  | 0.48 | Japanese Geotecnical Society-Procedures | Cubrinovski and Ishiara (2002) |
| 119 | Syncrude Oil Sand Tailings |  |  |  | 3.5 | 0.207 |  | 0.90 | 0.54 | 0.35 | NA | Jefferies and Been (2006), Golder Project Files |
| 120 | Isserk |  |  |  | 2 | 0.210 |  | 0.76 | 0.52 | 0.24 | NA | Jefferies and Been (2006), Golder Project Files |
| 121 | 1O6 Crushed Sand |  | 0.3 | 0.7 |  | 0.210 | 2.800 | 0.77 |  |  | ASTM C136, D4254, and D1557 | Zheng and Hryciw (2016), Cho et al. (2006) |
| 122 | Isserk |  |  |  | 5 | 0.210 |  | 0.83 | 0.55 | 0.28 | NA | Jefferies and Been (2006), Golder Project Files |
| 123 | Ottawa #60/80 Sand |  | 0.65 | 0.78 |  | 0.210 | 2.400 | 0.85 | 0.55 | 0.30 | NA | Zheng and Hryciw (2016), Sukumaran and Ashmawy (2001) |
| 124 | Isserk |  |  |  | 10 | 0.210 |  | 0.86 | 0.44 | 0.42 | NA | Jefferies and Been (2006), Golder Project Files |
| 125 | Class IIA, Michigan |  | 0.62 | 0.69 |  | 0.210 | 1.900 | 0.86 | 0.56 | 0.30 | ASTM D 4254 and ASTM D 4253 | Zheng and Hryciw (2016) |
| 126 | Fontainebleau Sand |  | 0.65 | 0.71 |  | 0.210 | 1.200 | 0.87 | 0.54 | 0.33 | Procedure proposed by Kolbuszewski (1948) | Zheng and Hryciw (2016), Tsomokos and Georgiannou (2010) |
| 127 | Toyoura |  |  |  | 0 | 0.210 |  | 0.87 | 0.66 | 0.22 | NA | Jefferies and Been (2006), Golder Project Files |
| 128 | Fontainebleau Sand |  | 0.45 | 0.75 |  | 0.210 | 1.500 | 0.90 | 0.51 | 0.39 | NA | Zheng and Hryciw (2016), Yang et al. (2010) |
| 129 | Nevada Sand 50/80 | SP |  |  | 0 | 0.211 | 1.222 | 0.86 | 0.58 | 0.28 | Japanese Geotecnical Society-Procedures | Cubrinovski and Ishiara (2002), Lade et al. (1998) |
| 130 | Sands with Fines (5<FC<15%) |  |  |  |  | 0.212 |  |  |  | 0.45 | Japanese Geotecnical Society-Procedures | Cubrinovski and Ishiara (2002) |
| 131 | Clean Sands (FC=0-5%) |  |  |  |  | 0.215 |  |  |  | 0.44 | Japanese Geotecnical Society-Procedures | Cubrinovski and Ishiara (2002) |
| 132 | Sands with Clay (15<FC<30 %) |  |  |  |  | 0.216 |  |  |  | 0.66 | Japanese Geotecnical Society-Procedures | Cubrinovski and Ishiara (2002) |
| 133 | Clean Sands (FC=0-5%) |  |  |  |  | 0.219 |  |  |  | 0.41 | Japanese Geotecnical Society-Procedures | Cubrinovski and Ishiara (2002) |
| 134 | Ottawa 90 |  |  |  |  | 0.220 | 1.850 | 0.79 | 0.49 | 0.30 | NA | Mayne and Kulhawy (1991), Schmertmann (1978) |
| 135 | P3-S7 |  | 0.46 |  |  | 0.220 | 1.800 | 0.80 | 0.51 | 0.29 | ASTM D 4254 and ASTM D 4253 | Zheng and Hryciw (2016), Bareither et al. (2008) |
| 136 | Ottawa 50–70 |  | 0.7 | 0.8 |  | 0.220 | 1.100 | 0.84 | 0.57 | 0.27 | NA | Zheng and Hryciw (2016), Thomann (1990) |
| 137 | Sands with Clay (15<FC<30 %) |  |  |  |  | 0.220 |  |  |  | 0.60 | Japanese Geotecnical Society-Procedures | Cubrinovski and Ishiara (2002) |
| 138 | Sands with Clay (15<FC<30 %) |  |  |  |  | 0.222 |  |  |  | 0.56 | Japanese Geotecnical Society-Procedures | Cubrinovski and Ishiara (2002) |
| 139 | Clean Sands (FC=0-5%) |  |  |  |  | 0.228 |  |  |  | 0.39 | Japanese Geotecnical Society-Procedures | Cubrinovski and Ishiara (2002) |
| 140 | Douglas Lake Sand |  | 0.45 | 0.75 |  | 0.230 | 2.400 | 0.83 | 0.54 | 0.29 | NA | Zheng and Hryciw (2016), Thomann (1990) |
| 141 | Meghna Sand |  | 0.2 | 0.74 |  | 0.230 | 1.900 | 0.97 | 0.66 | 0.31 | NA | Zheng and Hryciw (2016), Yasin and Safiullah (2003) |
| 142 | Daytona Beach Sand |  | 0.3 | 0.7 |  | 0.230 | 1.400 | 1.00 | 0.64 | 0.36 | NA | Zheng and Hryciw (2016), Sukumaran and Ashmawy (2001) |
| 143 | Osterberg B3-S2 |  |  |  |  | 0.230 | 2.450 | 1.22 | 0.61 | 0.61 | Japanese Standards (Ishihara et al. 1978) | Ishiara et al (1979) |
| 144 | Sands with Fines (5<FC<15%) |  |  |  |  | 0.232 |  |  |  | 0.63 | Japanese Geotecnical Society-Procedures | Cubrinovski and Ishiara (2002) |
| 145 | Sands with Clay (15<FC<30 %) |  |  |  |  | 0.235 |  |  |  | 0.59 | Japanese Geotecnical Society-Procedures | Cubrinovski and Ishiara (2002) |
| ***Data #*** | ***Soil Type*** | ***Soil Classification*** | ***R*** | ***S*** | ***FC*** | ***D_50_ (mm)*** | ***Cu*** | ***e_max_*** | ***e_min_*** | ***e_max_-e_min_*** | ***Test Methodology*** | ***Reference*** |
| 146 | Clean Sands (FC=0-5%) |  |  |  |  | 0.236 |  |  |  | 0.42 | Japanese Geotecnical Society-Procedures | Cubrinovski and Ishiara (2002) |
| 147 | Reid Bedford |  |  |  | 0 | 0.240 |  | 0.87 | 0.55 | 0.32 | NA | Jefferies and Been (2006), Golder Project Files |
| 148 | Reid Bedford |  | 0.29 | 0.76 |  | 0.240 | 1.700 | 0.87 | 0.55 | 0.32 | NA | Mayne and Kulhawy (1991), Schmertmann (1978) |
| 149 | Kawagishi-cho S-4 |  |  |  |  | 0.240 | 2.100 | 1.16 | 0.63 | 0.53 | Japanese Standards (Ishihara et al. 1978) | Ishiara and Koga (1981), Ishiara and Koga (1981) |
| 150 | Sands with Clay (15<FC<30 %) |  |  |  |  | 0.242 |  |  |  | 0.52 | Japanese Geotecnical Society-Procedures | Cubrinovski and Ishiara (2002) |
| 151 | Clean Sands (FC=0-5%) |  |  |  |  | 0.245 |  |  |  | 0.41 | Japanese Geotecnical Society-Procedures | Cubrinovski and Ishiara (2002) |
| 152 | 1O2 Crushed Sand |  | 0.25 | 0.8 |  | 0.250 | 2.900 | 0.83 |  |  | ASTM C136, D4254, and D1557 | Zheng and Hryciw (2016), Cho et al. (2006) |
| 153 | Lausitz Sand |  | 0.51 |  |  | 0.250 | 3.100 | 0.85 | 0.44 | 0.41 | NA | Zheng and Hryciw (2016), Herle and Gudehus (1999) |
| 154 | Treasure Island, California |  | 0.56 | 0.72 |  | 0.250 | 1.800 | 0.85 | 0.57 | 0.28 | NA | Zheng and Hryciw (2016), Zheng and Hryciw (2016) |
| 155 | Ottawa 50–70 |  | 0.52 | 0.9 |  | 0.250 | 1.200 | 0.89 | 0.53 | 0.36 | NA | Zheng and Hryciw (2016), Zelasko et al. (1975) |
| 156 | 6F5 Crushed Sand |  | 0.25 | 0.8 |  | 0.250 | 3.300 | 0.91 |  |  | ASTM C136, D4254, and D1557 | Zheng and Hryciw (2016), Cho et al. (2006) |
| 157 | Evanston Beach 50–70 |  | 0.41 | 0.73 |  | 0.250 | 1.200 | 0.92 | 0.54 | 0.38 | NA | Zheng and Hryciw (2016), Zelasko et al. (1975) |
| 158 | Host Sand A2 | SP |  |  | 2 | 0.250 | 1.625 | 0.98 | 0.60 | 0.38 | Non-Standard Procedures | Cubrinovski and Ishiara (2002), Thevanayagam (1998) |
| 159 | Franklin Falls 50–70 |  | 0.34 | 0.52 |  | 0.250 | 1.200 | 1.10 | 0.64 | 0.46 | NA | Zheng and Hryciw (2016), Zelasko et al. (1975) |
| 160 | Kawagishi-cho S-8 |  |  |  |  | 0.250 | 2.200 | 1.13 | 0.67 | 0.46 | Japanese Standards (Ishihara et al. 1978) | Ishiara and Koga (1981), Ishiara and Koga (1981) |
| 161 | Agsco 50–80 |  | 0.2 | 0.5 |  | 0.250 | 1.300 | 1.24 | 0.79 | 0.45 | NA | Zheng and Hryciw (2016), Thomann (1990) |
| 162 | Clean Sands (FC=0-5%) |  |  |  |  | 0.254 |  |  |  | 0.38 | Japanese Geotecnical Society-Procedures | Cubrinovski and Ishiara (2002) |
| 163 | 3C7 Crushed Sand |  | 0.25 | 0.8 |  | 0.260 | 3.200 | 0.85 |  |  | ASTM C136, D4254, and D1557 | Zheng and Hryciw (2016), Cho et al. (2006) |
| 164 | Chesterton, Indiana Dunes |  | 0.64 | 0.76 |  | 0.260 | 1.300 | 0.87 | 0.57 | 0.30 | NA | Zheng and Hryciw (2016) |
| 165 | Kawagishi-cho S-6 |  |  |  |  | 0.260 | 2.000 | 1.09 | 0.66 | 0.43 | Japanese Standards (Ishihara et al. 1978) | Ishiara and Koga (1981) |
| 166 | Sands with Clay (15<FC<30 %) |  |  |  |  | 0.261 |  |  |  | 0.55 | Japanese Geotecnical Society-Procedures | Cubrinovski and Ishiara (2002) |
| 167 | Clean Sands (FC=0-5%) |  |  |  |  | 0.263 |  |  |  | 0.33 | Japanese Geotecnical Society-Procedures | Cubrinovski and Ishiara (2002) |
| 168 | Clean Sands (FC=0-5%) |  |  |  |  | 0.263 |  |  |  | 0.42 | Japanese Geotecnical Society-Procedures | Cubrinovski and Ishiara (2002) |
| 169 | Sands with Clay (15<FC<30 %) |  |  |  |  | 0.264 |  |  |  | 0.63 | Japanese Geotecnical Society-Procedures | Cubrinovski and Ishiara (2002) |
| 170 | Sands with Clay (15<FC<30 %) |  |  |  |  | 0.269 |  |  |  | 0.57 | Japanese Geotecnical Society-Procedures | Cubrinovski and Ishiara (2002) |
| 171 | Bennett Silty Sand a |  |  |  | 34 | 0.270 |  | 0.68 | 0.18 | 0.50 | NA | Jefferies and Been (2006), Golder Project Files |
| 172 | P2-S6 |  | 0.25 |  |  | 0.270 | 3.800 | 0.76 | 0.46 | 0.30 | ASTM D 4254 and ASTM D 4253 | Zheng and Hryciw (2016), Bareither et al. (2008) |
| 173 | Nerlerk |  |  |  | 1.9 | 0.270 |  | 0.81 | 0.54 | 0.28 | NA | Jefferies and Been (2006), Golder Project Files |
| 174 | 3P3 Crushed Sand |  | 0.2 | 0.7 |  | 0.270 | 2.200 | 0.95 |  |  | ASTM C136, D4254, and D1557 | Zheng and Hryciw (2016), Cho et al. (2006) |
| ***Data #*** | ***Soil Type*** | ***Soil Classification*** | ***R*** | ***S*** | ***FC*** | ***D_50_ (mm)*** | ***Cu*** | ***e_max_*** | ***e_min_*** | ***e_max_-e_min_*** | ***Test Methodology*** | ***Reference*** |
| 175 | Ottawa #90 Sand |  | 0.16 | 0.6 |  | 0.270 | 2.200 | 1.10 | 0.73 | 0.37 | NA | Zheng and Hryciw (2016), Sukumaran and Ashmawy (2001) |
| 176 | Clean Sands (FC=0-5%) |  |  |  |  | 0.273 |  |  |  | 0.36 | Japanese Geotecnical Society-Procedures | Cubrinovski and Ishiara (2002) |
| 177 | Sands with Clay (15<FC<30 %) |  |  |  |  | 0.274 |  |  |  | 0.49 | Japanese Geotecnical Society-Procedures | Cubrinovski and Ishiara (2002) |
| 178 | Sands with Fines (5<FC<15%) |  |  |  |  | 0.278 |  |  |  | 0.50 | Japanese Geotecnical Society-Procedures | Cubrinovski and Ishiara (2002) |
| 179 | Sands with Clay (15<FC<30 %) |  |  |  |  | 0.279 |  |  |  | 0.61 | Japanese Geotecnical Society-Procedures | Cubrinovski and Ishiara (2002) |
| 180 | 2L6 Crushed Sand |  | 0.25 | 0.8 |  | 0.280 | 3.500 | 0.84 |  |  | ASTM C136, D4254, and D1557 | Zheng and Hryciw (2016), Cho et al. (2006) |
| 181 | Ottawa |  |  |  |  | 0.280 | 1.100 | 0.87 | 0.55 | 0.32 | NA | Mayne and Kulhawy (1991), Lambrechts and Leonards (1978) |
| 182 | Kogyuk |  |  |  | 5 | 0.280 |  | 0.87 | 0.56 | 0.31 | NA | Jefferies and Been (2006), Golder Project Files |
| 183 | Ham River |  | 0.45 | 0.65 |  | 0.280 | 1.600 | 0.92 | 0.59 | 0.33 | NA | Zheng and Hryciw (2016), Coop and Lee (1993) |
| 184 | Nerlerk |  |  |  | 2 | 0.280 |  | 0.94 | 0.62 | 0.32 | NA | Jefferies and Been (2006), Sladen et al. (1985) |
| 185 | Nerlerk |  | 0.43 | 0.75 |  | 0.280 | 2.000 | 0.94 | 0.62 | 0.32 | NA | Zheng and Hryciw (2016), Sladen et al. (1985) |
| 186 | Nerlerk |  |  |  | 12 | 0.280 |  | 0.96 | 0.43 | 0.53 | NA | Jefferies and Been (2006), Sladen et al. (1985) |
| 187 | Castro Sand C |  |  |  | 0 | 0.280 |  | 0.99 | 0.66 | 0.33 | NA | Jefferies and Been (2006), Castro (1969) |
| 188 | Showa Bridge S-1 |  |  |  |  | 0.280 | 2.400 | 1.10 | 0.65 | 0.45 | Japanese Standards (Ishihara et al. 1978) | Ishiara and Koga (1981) |
| 189 | Niigata South Bank | SP |  |  |  | 0.280 | 2.400 | 1.10 | 0.65 | 0.45 | Japanese Society for Soil Mechanics and Foundation Engineering, 1979 | Skempton (1986) |
| 190 | Osterberg O-4 |  |  |  |  | 0.280 | 4.100 | 1.11 | 0.59 | 0.51 | Japanese Standards (Ishihara et al. 1978) | Ishiara and Koga (1981) |
| 191 | Osterberg O-7 |  |  |  |  | 0.280 | 2.400 | 1.14 | 0.67 | 0.47 | Japanese Standards (Ishihara et al. 1978) | Ishiara and Koga (1981) |
| 192 | Kawagishi-cho S-9 |  |  |  |  | 0.280 | 1.900 | 1.14 | 0.71 | 0.43 | Japanese Standards (Ishihara et al. 1978) | Ishiara and Koga (1981) |
| 193 | Osterberg B3-S4/2 |  |  |  |  | 0.280 | 1.580 | 1.15 | 0.61 | 0.53 | Japanese Standards (Ishihara et al. 1978) | Ishiara et al (1979) |
| 194 | Niigata Road Site D=8.4 m/2 | SP |  |  |  | 0.280 | 1.600 | 1.15 | 0.61 | 0.54 | Japanese Society for Soil Mechanics and Foundation Engineering, 1979 | Skempton (1986) |
| 195 | Niigata Station D=9.6 m | SP |  |  |  | 0.280 |  | 1.20 | 0.75 | 0.45 | Japanese Society for Soil Mechanics and Foundation Engineering, 1979 | Skempton (1986) |
| 196 | P3-S6 |  | 0.36 |  |  | 0.290 | 2.100 | 0.77 | 0.50 | 0.27 | ASTM D 4254 and ASTM D 4253 | Zheng and Hryciw (2016), Bareither et al. (2008) |
| 197 | P1-S7 |  | 0.42 |  |  | 0.290 | 2.000 | 0.81 | 0.52 | 0.29 | ASTM D 4254 and ASTM D 4253 | Zheng and Hryciw (2016), Bareither et al. (2008) |
| 198 | Showa Bridge O-1 |  |  |  |  | 0.290 | 1.500 | 1.07 | 0.63 | 0.44 | Japanese Standards (Ishihara et al. 1978) | Ishiara and Koga (1981) |
| 199 | Niigata South Bank/2 | SP |  |  |  | 0.290 | 1.500 | 1.08 | 0.64 | 0.44 | Japanese Society for Soil Mechanics and Foundation Engineering, 1979 | Skempton (1986) |
| ***Data #*** | ***Soil Type*** | ***Soil Classification*** | ***R*** | ***S*** | ***FC*** | ***D_50_ (mm)*** | ***Cu*** | ***e_max_*** | ***e_min_*** | ***e_max_-e_min_*** | ***Test Methodology*** | ***Reference*** |
| 200 | Showa Bridge O-2 |  |  |  |  | 0.290 | 1.400 | 1.08 | 0.63 | 0.45 | Japanese Standards (Ishihara et al. 1978) | Ishiara and Koga (1981) |
| 201 | Showa Bridge O-3 |  |  |  |  | 0.290 | 1.400 | 1.09 | 0.66 | 0.43 | Japanese Standards (Ishihara et al. 1978) | Ishiara and Koga (1981) |
| 202 | Niigata Station D=9.5 m | SP |  |  |  | 0.290 |  | 1.21 | 0.76 | 0.45 | Japanese Society for Soil Mechanics and Foundation Engineering, 1979 | Skempton (1986) |
| 203 | Niigata Station D=9.25 m | SP |  |  |  | 0.290 |  | 1.22 | 0.76 | 0.46 | Japanese Society for Soil Mechanics and Foundation Engineering, 1979 | Skempton (1986) |
| 204 | Amauligak I-65 |  |  |  | 3 | 0.290 |  |  |  |  | NA | Jefferies and Been (2006), Golder Project Files |
| 205 | San Fernando 3 |  |  |  | 11 | 0.290 |  |  |  |  | NA | Jefferies and Been (2006), Seed et al. (1988) |
| 206 | Clean Sands (FC=0-5%) |  |  |  |  | 0.294 |  |  |  | 0.36 | Japanese Geotecnical Society-Procedures | Cubrinovski and Ishiara (2002) |
| 207 | Clean Sands (FC=0-5%) |  |  |  |  | 0.299 |  |  |  | 0.40 | Japanese Geotecnical Society-Procedures | Cubrinovski and Ishiara (2002) |
| 208 | P1-S4 |  | 0.59 |  |  | 0.300 | 2.700 | 0.70 | 0.40 | 0.30 | ASTM D 4254 and ASTM D 4253 | Zheng and Hryciw (2016), Bareither et al. (2008) |
| 209 | Evanston Beach Sand |  | 0.75 | 0.8 |  | 0.300 | 1.800 | 0.79 | 0.50 | 0.29 | NA | Zheng and Hryciw (2016), Baxter and Mitchell (2004) |
| 210 | 7U7 Crushed Sand |  | 0.2 | 0.8 |  | 0.300 | 3.200 | 0.79 |  |  | ASTM C136, D4254, and D1557 | Zheng and Hryciw (2016), Cho et al. (2006) |
| 211 | P2-S1 |  | 0.31 |  |  | 0.300 | 1.900 | 0.80 | 0.51 | 0.29 | ASTM D 4254 and ASTM D 4253 | Zheng and Hryciw (2016), Bareither et al. (2008) |
| 212 | Michigan Dunes |  | 0.62 | 0.72 |  | 0.300 | 1.500 | 0.85 | 0.56 | 0.29 | NA | Zheng and Hryciw (2016) |
| 213 | Sydney Sand |  |  |  | 10 | 0.300 | 1.500 | 0.86 | 0.57 | 0.29 | NA | Bobei et al. (2009) |
| 214 | Ham River Sand |  | 0.55 | 0.75 |  | 0.300 | 1.300 | 0.87 | 0.53 | 0.34 | Procedure proposed by Kolbuszewski (1948) | Zheng and Hryciw (2016), Tsomokos and Georgiannou (2010) |
| 215 | M31 Sand |  | 0.75 | 0.7 |  | 0.300 | 1.300 | 0.87 | 0.53 | 0.34 | Procedure proposed by Kolbuszewski (1948) | Zheng and Hryciw (2016), Tsomokos and Georgiannou (2010) |
| 216 | M31 Sand |  | 0.62 | 0.7 |  | 0.300 | 1.600 | 0.87 | 0.53 | 0.34 | NA | Zheng and Hryciw (2016), Georgiannou and Konstadinou (2013) |
| 217 | Lone Star 60 |  |  |  |  | 0.300 | 1.480 | 0.91 | 0.57 | 0.34 | NA | Mayne and Kulhawy (1991), Villet and Mitchell (1981) |
| 218 | Ogishima Island |  |  |  |  | 0.300 | 4.000 | 1.08 | 0.57 | 0.51 | Japanese Society for Soil Mechanics and Foundation Engineering, 1979 | Skempton (1986) |
| 219 | Fraser River Sand |  | 0.43 | 0.5 |  | 0.300 | 1.900 | 1.13 | 0.78 | 0.35 | NA | Zheng and Hryciw (2016), Sukumaran and Ashmawy (2001) |
| 220 | Niigata Road Site D=8.4 m | SP |  |  |  | 0.300 | 1.800 | 1.15 | 0.59 | 0.56 | Japanese Society for Soil Mechanics and Foundation Engineering, 1979 | Skempton (1986) |
| 221 | Osterberg B3-S4 |  |  |  |  | 0.300 | 1.830 | 1.16 | 0.59 | 0.57 | Japanese Standards (Ishihara et al. 1978) | Ishiara et al (1979) |
| 222 | Niigata Road Site | SP |  |  |  | 0.300 | 1.700 | 1.16 | 0.64 | 0.52 | Japanese Society for Soil Mechanics and Foundation Engineering, 1979 | Skempton (1986) |
| ***Data #*** | ***Soil Type*** | ***Soil Classification*** | ***R*** | ***S*** | ***FC*** | ***D_50_ (mm)*** | ***Cu*** | ***e_max_*** | ***e_min_*** | ***e_max_-e_min_*** | ***Test Methodology*** | ***Reference*** |
| 223 | 1K9 Crushed Sand |  | 0.2 | 0.4 |  | 0.300 | 3.400 | 1.16 |  |  | ASTM C136, D4254, and D1557 | Zheng and Hryciw (2016), Cho et al. (2006) |
| 224 | Niigata Road Site D=9.3 m | SP |  |  |  | 0.300 | 1.800 | 1.18 | 0.68 | 0.50 | Japanese Society for Soil Mechanics and Foundation Engineering, 1979 | Skempton (1986) |
| 225 | Osterberg B3-S5/2 |  |  |  |  | 0.300 | 1.790 | 1.18 | 0.68 | 0.50 | Japanese Standards (Ishihara et al. 1978) | Ishiara et al (1979) |
| 226 | Niigata Station D=9.8 m | SP |  |  |  | 0.300 |  | 1.20 | 0.78 | 0.42 | Japanese Society for Soil Mechanics and Foundation Engineering, 1979 | Skempton (1986) |
| 227 | Clean Sands (FC=0-5%) |  |  |  |  | 0.305 |  |  |  | 0.42 | Japanese Geotecnical Society-Procedures | Cubrinovski and Ishiara (2002) |
| 228 | P1-S1 |  | 0.5 |  |  | 0.310 | 1.900 | 0.76 | 0.48 | 0.28 | ASTM D 4254 and ASTM D 4253 | Zheng and Hryciw (2016), Bareither et al. (2008) |
| 229 | Frankston |  |  |  |  | 0.310 | 2.050 | 0.79 | 0.46 | 0.33 | NA | Mayne and Kulhawy (1991), Chapman and Donald (1981) |
| 230 | P1-S3 |  | 0.4 |  |  | 0.310 | 2.300 | 0.83 | 0.50 | 0.33 | ASTM D 4254 and ASTM D 4253 | Zheng and Hryciw (2016), Bareither et al. (2008) |
| 231 | Oakland County, Michigan |  | 0.65 | 0.72 |  | 0.310 | 1.600 | 0.86 | 0.53 | 0.33 | NA | Zheng and Hryciw (2016) |
| 232 | Amauligak I-65 |  |  |  | 9 | 0.310 |  |  |  |  | NA | Jefferies and Been (2006), Golder Project Files |
| 233 | P2-S4 |  | 0.4 |  |  | 0.320 | 5.300 | 0.68 | 0.39 | 0.29 | ASTM D 4254 and ASTM D 4253 | Zheng and Hryciw (2016), Bareither et al. (2008) |
| 234 | Glass Beads |  |  |  | 0 | 0.320 | 1.370 | 0.72 | 0.54 | 0.18 | NA | Santamarina and Cho (2001) |
| 235 | Glass Beads |  | 1 | 1 |  | 0.320 | 1.400 | 0.72 | 0.54 | 0.18 | ASTM C136, D4254, and D1557 | Zheng and Hryciw (2016), Cho et al. (2006) |
| 236 | S. Oakleigh Medium |  |  |  |  | 0.320 | 2.200 | 0.75 | 0.41 | 0.34 | NA | Mayne and Kulhawy (1991), Veismanis (1974) |
| 237 | Erksak |  |  |  | 1 | 0.320 |  | 0.81 | 0.61 | 0.19 | NA | Jefferies and Been (2006), Golder Project Files |
| 238 | New Madrid, Missouri |  | 0.57 | 0.73 |  | 0.320 | 2.200 | 0.81 | 0.52 | 0.29 | NA | Zheng and Hryciw (2016) |
| 239 | 5U1 Crushed Sand |  | 0.15 | 0.7 |  | 0.320 | 3.500 | 0.84 |  |  | ASTM C136, D4254, and D1557 | Zheng and Hryciw (2016), Cho et al. (2006) |
| 240 | 8B8 Crushed Sand |  | 0.25 | 0.8 |  | 0.320 | 3.700 | 0.85 |  |  | ASTM C136, D4254, and D1557 | Zheng and Hryciw (2016), Cho et al. (2006) |
| 241 | Kawagishi-cho S-1 |  |  |  |  | 0.320 | 3.300 | 1.08 | 0.64 | 0.44 | Japanese Standards (Ishihara et al. 1978) | Ishiara and Koga (1981) |
| 242 | Clean Sands (FC=0-5%) |  |  |  |  | 0.328 |  |  |  | 0.35 | Japanese Geotecnical Society-Procedures | Cubrinovski and Ishiara (2002) |
| 243 | Clean Sands (FC=0-5%) |  |  |  |  | 0.328 |  |  |  | 0.39 | Japanese Geotecnical Society-Procedures | Cubrinovski and Ishiara (2002) |
| 244 | Earlston |  |  |  |  | 0.330 | 2.600 | 0.73 | 0.40 | 0.32 | NA | Mayne and Kulhawy (1991), Veismanis (1974) |
| 245 | Erksak |  |  |  | 0.7 | 0.330 |  | 0.75 | 0.52 | 0.23 | NA | Jefferies and Been (2006), Golder Project Files |
| 246 | 9F1 Crushed Sand |  | 0.2 | 0.8 |  | 0.330 | 3.500 | 0.90 |  |  | ASTM C136, D4254, and D1557 | Zheng and Hryciw (2016), Cho et al. (2006) |
| 247 | 6A2 Crushed Sand |  | 0.2 | 0.75 |  | 0.330 | 5.500 | 0.93 |  |  | ASTM C136, D4254, and D1557 | Zheng and Hryciw (2016), Cho et al. (2006) |
| 248 | Scotts Valley, California |  | 0.4 | 0.73 |  | 0.330 | 1.500 | 0.94 | 0.60 | 0.34 | NA | Zheng and Hryciw (2016) |
| 249 | 6H1 Crushed Sand |  | 0.2 | 0.8 |  | 0.330 | 3.800 | 0.97 |  |  | ASTM C136, D4254, and D1557 | Zheng and Hryciw (2016), Cho et al. (2006) |
| 250 | Sands with Fines (5<FC<15%) |  |  |  |  | 0.334 |  |  |  | 0.61 | Japanese Geotecnical Society-Procedures | Cubrinovski and Ishiara (2002) |
| ***Data #*** | ***Soil Type*** | ***Soil Classification*** | ***R*** | ***S*** | ***FC*** | ***D_50_ (mm)*** | ***Cu*** | ***e_max_*** | ***e_min_*** | ***e_max_-e_min_*** | ***Test Methodology*** | ***Reference*** |
| 251 | P1-S6 |  | 0.62 |  |  | 0.340 | 2.400 | 0.69 | 0.43 | 0.26 | ASTM D 4254 and ASTM D 4253 | Zheng and Hryciw (2016), Bareither et al. (2008) |
| 252 | Ackerman Lake Sand |  | 0.6 | 0.8 |  | 0.340 | 2.500 | 0.72 | 0.48 | 0.24 | NA | Zheng and Hryciw (2016), Thomann (1990) |
| 253 | Kogyuk Sand | SP |  |  | 10 | 0.340 | 2.300 | 0.93 | 0.47 | 0.46 | ASTM D2049 | Been and Jefferies (1985) |
| 254 | TS Sand | SP |  |  |  | 0.340 | 1.950 | 0.97 | 0.58 | 0.38 | ASTM | Kokusho and Yoshida (1997) |
| 255 | Osterberg B3-S1/2 |  |  |  |  | 0.340 | 1.800 | 1.10 | 0.66 | 0.44 | Japanese Standards (Ishihara et al. 1978) | Ishiara et al (1979) |
| 256 | Niigata Road Site D=9.2 m | SP |  |  |  | 0.340 | 1.400 | 1.16 | 0.67 | 0.49 | Japanese Society for Soil Mechanics and Foundation Engineering, 1979 | Skempton (1986) |
| 257 | Osterberg B3-S5 |  |  |  |  | 0.340 | 1.440 | 1.16 | 0.67 | 0.49 | Japanese Standards (Ishihara et al. 1978) | Ishiara et al (1979) |
| 258 | Clean Sands (FC=0-5%) |  |  |  |  | 0.346 |  |  |  | 0.58 | Japanese Geotecnical Society-Procedures | Cubrinovski and Ishiara (2002) |
| 259 | Clean Sands (FC=0-5%) |  |  |  |  | 0.346 |  |  |  | 0.37 | Japanese Geotecnical Society-Procedures | Cubrinovski and Ishiara (2002) |
| 260 | Sands with Fines (5<FC<15%) |  |  |  |  | 0.346 |  |  |  | 0.50 | Japanese Geotecnical Society-Procedures | Cubrinovski and Ishiara (2002) |
| 261 | Kogyuk Sand | SP |  |  | 0 | 0.350 | 1.700 | 0.78 | 0.52 | 0.26 | ASTM D2049 | Been and Jefferies (1985) |
| 262 | ASTM graded Sand |  |  |  | 0 | 0.350 | 1.650 | 0.82 | 0.50 | 0.32 | NA | Santamarina and Cho (2001) |
| 263 | ASTM graded Sand |  | 0.8 | 0.9 |  | 0.350 | 1.700 | 0.82 | 0.50 | 0.32 | ASTM C136, D4254, and D1557 | Zheng and Hryciw (2016), Cho et al. (2006) |
| 264 | Kogyuk Sand | SP |  |  | 2 | 0.350 | 1.800 | 0.83 | 0.47 | 0.36 | ASTM D2049 | Been and Jefferies (1985) |
| 265 | Kogyuk |  |  |  | 2 | 0.350 |  | 0.83 | 0.47 | 0.36 | NA | Jefferies and Been (2006), Golder Project Files |
| 266 | Sydney Sand | SP |  |  |  | 0.350 | 1.650 | 0.84 | 0.56 | 0.28 | NA | Rix and Stokoe (1991) |
| 267 | Kogyuk |  |  |  | 5 | 0.350 |  | 0.87 | 0.49 | 0.38 | NA | Jefferies and Been (2006), Golder Project Files |
| 268 | Capitola, California |  | 0.48 | 0.72 |  | 0.350 | 1.600 | 0.89 | 0.57 | 0.32 | NA | Zheng and Hryciw (2016) |
| 269 | Kogyuk |  |  |  | 10 | 0.350 |  | 0.93 | 0.46 | 0.47 | NA | Jefferies and Been (2006), Golder Project Files |
| 270 | Erksak |  |  |  |  | 0.350 | 2.200 | 0.96 | 0.53 | 0.44 | NA | Mayne and Kulhawy (1991), Been et al. (1987) |
| 271 | Hostun RF Sand |  | 0.3 |  |  | 0.350 | 1.700 | 0.98 | 0.61 | 0.37 | NA | Zheng and Hryciw (2016), Herle and Gudehus (1999) |
| 272 | Hostun Fine |  |  |  |  | 0.350 | 2.220 | 1.00 | 0.65 | 0.35 | NA | Mayne and Kulhawy (1991), Canou et al. (1988) |
| 273 | Kawagishi-cho S-3 |  |  |  |  | 0.350 | 2.800 | 1.07 | 0.65 | 0.41 | Japanese Standards (Ishihara et al. 1978) | Ishiara and Koga (1981) |
| 274 | Niigata LD3-S3 |  |  |  |  | 0.350 | 2.000 | 1.07 | 0.57 | 0.50 | Japanese Standards | Ishiara et al (1978) |
| 275 | Kawagishi-cho S-5 |  |  |  |  | 0.350 | 2.100 | 1.07 | 0.66 | 0.42 | Japanese Standards (Ishihara et al. 1978) | Ishiara and Koga (1981) |
| 276 | Osterberg O-6 |  |  |  |  | 0.350 | 2.400 | 1.08 | 0.65 | 0.43 | Japanese Standards (Ishihara et al. 1978) | Ishiara and Koga (1981) |
| 277 | Kawagishi-cho |  |  |  |  | 0.350 | 2.400 | 1.08 | 0.63 | 0.45 | Japanese Society for Soil Mechanics and Foundation Engineering, 1979 | Skempton (1986) |
| 278 | Osterberg B3-S6/2 |  |  |  |  | 0.350 | 1.540 | 1.08 | 0.63 | 0.46 | Japanese Standards (Ishihara et al. 1978) | Ishiara et al (1979) |
| 279 | Clean Sands (FC=0-5%) |  |  |  |  | 0.353 |  |  |  | 0.43 | Japanese Geotecnical Society-Procedures | Cubrinovski and Ishiara (2002) |
| 280 | Erksak Sand |  |  |  | 3 | 0.355 |  | 0.96 | 0.53 | 0.44 | NA | Jefferies and Been (2006), Golder Project Files |
| ***Data #*** | ***Soil Type*** | ***Soil Classification*** | ***R*** | ***S*** | ***FC*** | ***D_50_ (mm)*** | ***Cu*** | ***e_max_*** | ***e_min_*** | ***e_max_-e_min_*** | ***Test Methodology*** | ***Reference*** |
| 281 | Sands with Fines (5<FC<15%) |  |  |  |  | 0.359 |  |  |  | 0.55 | Japanese Geotecnical Society-Procedures | Cubrinovski and Ishiara (2002) |
| 282 | Sandboil Sand |  |  |  | 3 | 0.360 | 2.400 | 0.79 | 0.51 | 0.28 | NA | Santamarina and Cho (2001) |
| 283 | Sandboil Sand |  | 0.55 | 0.7 |  | 0.360 | 2.410 | 0.79 | 0.51 | 0.28 | ASTM C136, D4254, and D1557 | Zheng and Hryciw (2016), Cho et al. (2006) |
| 284 | Rincon, New Mexico |  | 0.55 | 0.82 |  | 0.360 | 3.000 | 0.80 | 0.51 | 0.29 | NA | Zheng and Hryciw (2016) |
| 285 | Kogyuk Sand | SP |  |  | 5 | 0.360 | 2.000 | 0.87 | 0.49 | 0.38 | ASTM D2049 | Been and Jefferies (1985) |
| 286 | Niigata LD4-S2 |  |  |  |  | 0.360 | 2.700 | 1.02 | 0.65 | 0.37 | Japanese Standards | Ishiara et al (1978) |
| 287 | Osterberg B3-S6 |  |  |  |  | 0.360 | 1.460 | 1.07 | 0.62 | 0.45 | Japanese Standards (Ishihara et al. 1978) | Ishiara et al (1979) |
| 288 | Osterberg B3-S3 |  |  |  |  | 0.360 | 1.500 | 1.08 | 0.57 | 0.51 | Japanese Standards (Ishihara et al. 1978) | Ishiara et al (1979) |
| 289 | Bennett Silty Sand b |  |  |  | 26 | 0.370 |  | 0.52 | 0.33 | 0.19 | NA | Jefferies and Been (2006), Golder Project Files |
| 290 | Leighton Buzzard |  |  |  |  | 0.370 | 1.500 | 0.82 | 0.49 | 0.33 | NA | Mayne and Kulhawy (1991), Chong (1988) |
| 291 | Monterey 0 |  | 0.35 | 0.8 |  | 0.370 | 1.600 | 0.82 | 0.54 | 0.28 | NA | Mayne and Kulhawy (1991), Huntsman et al. (1986) |
| 292 | Monterey Sand |  |  |  | 0 | 0.370 |  | 0.82 | 0.54 | 0.28 | NA | Jefferies and Been (2006), Golder Project Files |
| 293 | Niigata LD4-S3 |  |  |  |  | 0.370 | 2.500 | 1.07 | 0.62 | 0.45 | Japanese Standards | Ishiara et al (1978) |
| 294 | Ottowa Sand | SP |  |  |  | 0.373 | 1.400 | 0.76 | 0.40 | 0.36 | ASTM D 2049-69 | Youd (1973) |
| 295 | Crushed Basalt |  |  |  |  | 0.378 | 1.400 | 1.30 | 0.68 | 0.62 | ASTM D 2049-69 | Youd (1973) |
| 296 | Del Monte white Sand |  |  |  |  | 0.378 | 1.400 | 0.95 | 0.50 | 0.46 | ASTM D 2049-69 | Youd (1973) |
| 297 | P1-S2 |  | 0.61 |  |  | 0.380 | 2.200 | 0.67 | 0.42 | 0.25 | ASTM D 4254 and ASTM D 4253 | Zheng and Hryciw (2016), Bareither et al. (2008) |
| 298 | Sand O |  | 0.41 | 0.93 |  | 0.380 | 1.800 | 0.81 | 0.50 | 0.31 | ASTM D4253-93 and ASTM D4254-91 | Zheng and Hryciw (2016), Guo and Su (2007) |
| 299 | 8M8 Crushed Sand |  | 0.2 | 0.7 |  | 0.380 | 3.300 | 0.97 |  |  | ASTM C136, D4254, and D1557 | Zheng and Hryciw (2016), Cho et al. (2006) |
| 300 | Osterberg B3-S3/2 |  |  |  |  | 0.380 | 1.620 | 1.06 | 0.57 | 0.49 | Japanese Standards (Ishihara et al. 1978) | Ishiara et al (1979) |
| 301 | Clean Sands (FC=0-5%) |  |  |  |  | 0.387 |  |  |  | 0.37 | Japanese Geotecnical Society-Procedures | Cubrinovski and Ishiara (2002) |
| 302 | Sands with Fines (5<FC<15%) |  |  |  |  | 0.387 |  |  |  | 0.48 | Japanese Geotecnical Society-Procedures | Cubrinovski and Ishiara (2002) |
| 303 | Ottawa Sand | SP |  |  | 0 | 0.390 | 1.630 | 0.78 | 0.48 | 0.30 | ASTM | Cubrinovski and Ishiara (2002), Salgado et al. (2000) |
| 304 | Lone Star 30 |  |  |  |  | 0.390 | 1.860 | 0.82 | 0.54 | 0.29 | NA | Mayne and Kulhawy (1991), Villet and Mitchell (1981) |
| 305 | Ottawa Sand C-109 | SP |  |  | 0 | 0.390 | 1.952 | 0.83 | 0.50 | 0.33 | NA | Cubrinovski and Ishiara (2002), Pitman et al. (1994) |
| 306 | Hokksund |  |  |  |  | 0.390 | 2.200 | 0.88 | 0.54 | 0.34 | NA | Mayne and Kulhawy (1991), Baldi et al. (1981) |
| 307 | Hokksund Sand |  |  |  |  | 0.390 | 2.100 | 0.89 | 0.55 | 0.35 | NA | Huang Report (1991), Borden (1991) |
| 308 | Hokksund |  |  |  | 0 | 0.390 |  | 0.91 | 0.55 | 0.36 | NA | Jefferies and Been (2006), Golder Project Files |
| 309 | Osterberg B1-S7 |  |  |  |  | 0.390 | 1.720 | 0.93 | 0.51 | 0.42 | Japanese Standards (Ishihara et al. 1978) | Ishiara et al (1979) |
| 310 | Osterberg O-1 |  |  |  |  | 0.390 | 3.300 | 1.05 | 0.62 | 0.43 | Japanese Standards (Ishihara et al. 1978) | Ishiara and Koga (1981) |
| 311 | Niigata Road Site D=13.8 m | SP |  |  |  | 0.390 | 1.800 | 1.07 | 0.58 | 0.49 | Japanese Society for Soil Mechanics and Foundation Engineering, 1979 | Skempton (1986) |
| ***Data #*** | ***Soil Type*** | ***Soil Classification*** | ***R*** | ***S*** | ***FC*** | ***D_50_ (mm)*** | ***Cu*** | ***e_max_*** | ***e_min_*** | ***e_max_-e_min_*** | ***Test Methodology*** | ***Reference*** |
| 312 | Osterberg B3-S8 |  |  |  |  | 0.390 | 1.830 | 1.07 | 0.58 | 0.49 | Japanese Standards (Ishihara et al. 1978) | Ishiara et al (1979) |
| 313 | Clean Sands (FC=0-5%) |  |  |  |  | 0.394 |  |  |  | 0.39 | Japanese Geotecnical Society-Procedures | Cubrinovski and Ishiara (2002) |
| 314 | Lanchester 25/52 |  |  |  |  | 0.400 | 1.400 | 0.82 | 0.56 | 0.26 | NA | Mayne and Kulhawy (1991), Thomas (1968) |
| 315 | Karlsruhe Sand |  | 0.45 |  |  | 0.400 | 1.900 | 0.84 | 0.53 | 0.31 | NA | Zheng and Hryciw (2016), Herle and Gudehus (1999) |
| 316 | Muskegon, Michigan |  | 0.55 | 0.74 |  | 0.400 | 1.600 | 0.84 | 0.56 | 0.28 | NA | Zheng and Hryciw (2016) |
| 317 | 5Z9 Crushed Sand |  | 0.3 | 0.9 |  | 0.400 | 3.600 | 0.89 |  |  | ASTM C136, D4254, and D1557 | Zheng and Hryciw (2016), Cho et al. (2006) |
| 318 | Osterberg B1-S3 |  |  |  |  | 0.400 | 2.140 | 1.00 | 0.61 | 0.39 | Japanese Standards (Ishihara et al. 1978) | Ishiara et al (1979) |
| 319 | Osterberg B1-S4 |  |  |  |  | 0.400 | 1.600 | 1.01 | 0.62 | 0.38 | Japanese Standards (Ishihara et al. 1978) | Ishiara et al (1979) |
| 320 | Osterberg LD3-S10/2 |  |  |  |  | 0.400 | 2.000 | 1.02 | 0.62 | 0.39 | Japanese Standards (Ishihara et al. 1978) | Ishiara et al (1979) |
| 321 | Clean Sands (FC=0-5%) |  |  |  |  | 0.401 |  |  |  | 0.34 | Japanese Geotecnical Society-Procedures | Cubrinovski and Ishiara (2002) |
| 322 | Bennett Silty Sand c |  |  |  | 20 | 0.410 |  | 0.51 | 0.34 | 0.17 | NA | Jefferies and Been (2006), Golder Project Files |
| 323 | Osterberg LD3-S10 |  |  |  |  | 0.410 | 1.770 | 1.02 | 0.55 | 0.47 | Japanese Standards (Ishihara et al. 1978) | Ishiara et al (1979) |
| 324 | Clean Sands (FC=0-5%) |  |  |  |  | 0.416 |  |  |  | 0.41 | Japanese Geotecnical Society-Procedures | Cubrinovski and Ishiara (2002) |
| 325 | P2-S12 |  | 0.42 |  |  | 0.420 | 3.100 | 0.64 | 0.39 | 0.25 | ASTM D 4254 and ASTM D 4253 | Zheng and Hryciw (2016), Bareither et al. (2008) |
| 326 | Ottawa 35–45 |  | 0.6 | 0.9 |  | 0.420 | 1.200 | 0.82 | 0.48 | 0.34 | NA | Zheng and Hryciw (2016), Zelasko et al. (1975) |
| 327 | Evanston Beach 35–45 |  | 0.43 | 0.73 |  | 0.420 | 1.200 | 0.90 | 0.52 | 0.38 | NA | Zheng and Hryciw (2016), Zelasko et al. (1975) |
| 328 | Franklin Falls 35–45 |  | 0.35 | 0.52 |  | 0.420 | 1.200 | 1.04 | 0.63 | 0.41 | NA | Zheng and Hryciw (2016), Zelasko et al. (1975) |
| 329 | Osterberg O-2 |  |  |  |  | 0.420 | 2.100 | 1.05 | 0.64 | 0.41 | Japanese Standards (Ishihara et al. 1978) | Ishiara and Koga (1981) |
| 330 | Kawagishi-cho S-2 |  |  |  |  | 0.420 | 2.000 | 1.08 | 0.65 | 0.43 | Japanese Standards (Ishihara et al. 1978) | Ishiara and Koga (1981) |
| 331 | Clean Sands (FC=0-5%) |  |  |  |  | 0.424 |  |  |  | 0.54 | Japanese Geotecnical Society-Procedures | Cubrinovski and Ishiara (2002) |
| 332 | Gravelly Sands (FC<6 %) |  |  |  |  | 0.424 |  |  |  | 0.38 | Japanese Geotecnical Society-Procedures | Cubrinovski and Ishiara (2002) |
| 333 | Sands with Fines (5<FC<15%) |  |  |  |  | 0.424 |  |  |  | 0.48 | Japanese Geotecnical Society-Procedures | Cubrinovski and Ishiara (2002) |
| 334 | Hormuz Island |  | 0.2 | 0.5 |  | 0.430 | 3.200 | 1.05 | 0.72 | 0.33 | ASTM | Zheng and Hryciw (2016), Shahnazari and Rezvai (2013) |
| 335 | Clean Sands (FC=0-5%) |  |  |  |  | 0.431 |  |  |  | 0.33 | Japanese Geotecnical Society-Procedures | Cubrinovski and Ishiara (2002) |
| 336 | P1-S5 |  | 0.62 |  |  | 0.440 | 2.600 | 0.76 | 0.43 | 0.33 | ASTM D 4254 and ASTM D 4253 | Zheng and Hryciw (2016), Bareither et al. (2008) |
| 337 | Fort Davis, Texas |  | 0.41 | 0.68 |  | 0.440 | 8.600 | 0.85 | 0.51 | 0.34 | NA | Zheng and Hryciw (2016) |
| 338 | Hokksund |  |  |  |  | 0.440 | 2.200 | 0.91 | 0.54 | 0.37 | NA | Mayne and Kulhawy (1991), Lunne and Christoffersen (1985) & Parkin et al. (1980) |
| 339 | Clean Sands (FC=0-5%) |  |  |  |  | 0.448 |  |  |  | 0.34 | Japanese Geotecnical Society-Procedures | Cubrinovski and Ishiara (2002) |
| 340 | Monterey 0/30 |  |  |  |  | 0.450 | 1.370 | 0.80 | 0.56 | 0.24 | NA | Mayne and Kulhawy (1991), Sweenay (1987) |
| ***Data #*** | ***Soil Type*** | ***Soil Classification*** | ***R*** | ***S*** | ***FC*** | ***D_50_ (mm)*** | ***Cu*** | ***e_max_*** | ***e_min_*** | ***e_max_-e_min_*** | ***Test Methodology*** | ***Reference*** |
| 341 | Edgar |  |  |  |  | 0.450 | 1.790 | 0.92 | 0.54 | 0.38 | NA | Mayne and Kulhawy (1991), Veismanis (1974) & Holden (1976) |
| 342 | Niigata River Site D=7.5 m | SP |  |  | 1 | 0.450 | 2.100 | 0.97 | 0.55 | 0.42 | Japanese Society for Soil Mechanics and Foundation Engineering, 1979 | Skempton (1986) |
| 343 | Niigata LD4-S7 |  |  |  |  | 0.450 | 2.100 | 0.97 | 0.55 | 0.42 | Japanese Standards | Ishiara et al (1978) |
| 344 | Niigata Road Site/2 | SP |  |  |  | 0.450 | 1.800 | 1.03 | 0.56 | 0.47 | Japanese Society for Soil Mechanics and Foundation Engineering, 1979 | Skempton (1986) |
| 345 | Osterberg B3-S8/2 |  |  |  |  | 0.450 | 1.920 | 1.05 | 0.56 | 0.50 | Japanese Standards (Ishihara et al. 1978) | Ishiara et al (1979) |
| 346 | Leighton Buzzard 30%Mica |  |  |  | 0 | 0.450 |  | 1.79 | 0.82 | 0.97 | NA | Jefferies and Been (2006), Hird and Hassona (1990) |
| 347 | Niigata River Site D=7.5 m/2 | SP |  |  | 1 | 0.460 | 2.000 | 1.02 | 0.54 | 0.48 | Japanese Society for Soil Mechanics and Foundation Engineering, 1979 | Skempton (1986) |
| 348 | Niigata, River Site |  |  |  |  | 0.460 | 2.000 | 1.02 | 0.57 | 0.45 | Japanese Society for Soil Mechanics and Foundation Engineering, 1979 | Skempton (1986) |
| 349 | Osterberg B1-S6 |  |  |  |  | 0.460 | 1.890 | 1.05 | 0.54 | 0.51 | Japanese Standards (Ishihara et al. 1978) | Ishiara et al (1979) |
| 350 | Niigata LD3-S4 |  |  |  |  | 0.460 | 2.000 | 1.06 | 0.53 | 0.53 | Japanese Standards | Ishiara et al (1978) |
| 351 | Niigata River Site D=6.5 m | SP |  |  | 0 | 0.460 | 2.000 | 1.06 | 0.53 | 0.53 | Japanese Society for Soil Mechanics and Foundation Engineering, 1979 | Skempton (1986) |
| 352 | Osterberg O-3 |  |  |  |  | 0.470 | 2.100 | 1.02 | 0.62 | 0.40 | Japanese Standards (Ishihara et al. 1978) | Ishiara and Koga (1981) |
| 353 | Hoston Sand |  | 0.3 | 0.6 |  | 0.470 | 1.400 | 1.04 | 0.64 | 0.40 | NA | Zheng and Hryciw (2016), Ezaoui and Benedetto (2009) |
| 354 | Leighton Buzzard 17%Mica |  |  |  | 0 | 0.470 |  | 1.32 | 0.62 | 0.71 | NA | Jefferies and Been (2006), Hird and Hassona (1990) |
| 355 | P3-S5 |  | 0.56 |  |  | 0.480 | 3.000 | 0.62 | 0.38 | 0.24 | ASTM D 4254 and ASTM D 4253 | Zheng and Hryciw (2016), Bareither et al. (2008) |
| 356 | P3-S2 |  | 0.48 |  |  | 0.480 | 4.800 | 0.70 | 0.39 | 0.31 | ASTM D 4254 and ASTM D 4253 | Zheng and Hryciw (2016), Bareither et al. (2008) |
| 357 | P4-S2 |  | 0.31 |  |  | 0.480 | 2.900 | 0.72 | 0.44 | 0.28 | ASTM D 4254 and ASTM D 4253 | Zheng and Hryciw (2016), Bareither et al. (2008) |
| 358 | 2Z8 Crushed Sand |  | 0.1 | 0.6 |  | 0.480 | 5.000 | 0.86 |  |  | ASTM C136, D4254, and D1557 | Zheng and Hryciw (2016), Cho et al. (2006) |
| 359 | Osterberg B3-S7 |  |  |  |  | 0.480 | 1.670 | 0.99 | 0.55 | 0.44 | Japanese Standards (Ishihara et al. 1978) | Ishiara et al (1979) |
| 360 | Niigata Road Site D=12.8 m | SP |  |  |  | 0.480 | 1.700 | 0.99 | 0.55 | 0.44 | Japanese Society for Soil Mechanics and Foundation Engineering, 1979 | Skempton (1986) |
| 361 | Osterberg B3-S7/2 |  |  |  |  | 0.480 | 1.830 | 1.01 | 0.56 | 0.44 | Japanese Standards (Ishihara et al. 1978) | Ishiara et al (1979) |
| ***Data #*** | ***Soil Type*** | ***Soil Classification*** | ***R*** | ***S*** | ***FC*** | ***D_50_ (mm)*** | ***Cu*** | ***e_max_*** | ***e_min_*** | ***e_max_-e_min_*** | ***Test Methodology*** | ***Reference*** |
| 362 | Niigata Road Site D=12.8 m/2 | SP |  |  |  | 0.480 | 1.800 | 1.01 | 0.56 | 0.45 | Japanese Society for Soil Mechanics and Foundation Engineering, 1979 | Skempton (1986) |
| 363 | Margaret River Sand |  | 0.7 | 0.7 |  | 0.490 | 1.900 | 0.87 |  |  | ASTM C136, D4254, and D1557 | Zheng and Hryciw (2016), Cho et al. (2006) |
| 364 | Gravelly Sands (FC<6 %) |  |  |  |  | 0.499 |  |  |  | 0.36 | Japanese Geotecnical Society-Procedures | Cubrinovski and Ishiara (2002) |
| 365 | P2-S9 |  | 0.43 |  |  | 0.500 | 4.200 | 0.56 | 0.33 | 0.23 | ASTM D 4254 and ASTM D 4253 | Zheng and Hryciw (2016), Bareither et al. (2008) |
| 366 | P2-S8 |  | 0.37 |  |  | 0.500 | 3.100 | 0.64 | 0.40 | 0.24 | ASTM D 4254 and ASTM D 4253 | Zheng and Hryciw (2016), Bareither et al. (2008) |
| 367 | Density Sand |  | 0.8 | 0.9 |  | 0.500 | 1.900 | 0.72 | 0.48 | 0.24 | NA | Zheng and Hryciw (2016), Baxter and Mitchell (2004) |
| 368 | Leighton Buzzard |  |  |  | 0 | 0.500 |  | 0.79 | 0.52 | 0.28 | NA | Jefferies and Been (2006), Hird and Hassona (1990) |
| 369 | Michigan 2NS |  | 0.53 | 0.67 |  | 0.500 | 2.300 | 0.82 | 0.54 | 0.28 | NA | Zheng and Hryciw (2016) |
| 370 | Zbraslav Sand |  | 0.3 |  |  | 0.500 | 2.600 | 0.82 | 0.52 | 0.30 | NA | Zheng and Hryciw (2016), Herle and Gudehus (1999) |
| 371 | Ticino |  | 0.38 | 0.79 |  | 0.500 | 1.580 | 0.92 | 0.57 | 0.35 | NA | Mayne and Kulhawy (1991), Baldi et al. (1981), Baldi et al. (1986), Bellotti et al. (1979) & Canou et al. (1988) |
| 372 | Teesta Sand |  | 0.4 | 0.65 |  | 0.500 | 2.500 | 0.92 | 0.57 | 0.35 | NA | Zheng and Hryciw (2016), Yasin and Safiullah (2003) |
| 373 | Leighton Buzzard 10%Mica |  |  |  | 0 | 0.500 |  | 1.07 | 0.59 | 0.48 | NA | Jefferies and Been (2006), Hird and Hassona (1990) |
| 374 | 9C1 Crushed Sand |  | 0.25 | 0.7 |  | 0.520 | 2.300 | 0.91 |  |  | ASTM C136, D4254, and D1557 | Zheng and Hryciw (2016), Cho et al. (2006) |
| 375 | Osterberg O-5 |  |  |  |  | 0.520 | 2.100 | 1.05 | 0.65 | 0.40 | Japanese Standards (Ishihara et al. 1978) | Ishiara and Koga (1981) |
| 376 | Ottawa #20/70 Sand |  | 0.32 | 0.81 |  | 0.530 | 2.400 | 0.78 | 0.47 | 0.31 | NA | Zheng and Hryciw (2016), Sukumaran and Ashmawy (2001) |
| 377 | Ottawa |  |  |  | 0 | 0.530 |  | 0.79 | 0.49 | 0.30 | NA | Jefferies and Been (2006), Golder Project Files |
| 378 | Ticino-4 | SP |  |  | 0 | 0.530 |  | 0.89 | 0.60 | 0.29 | NA | Jefferies and Been (2006), Golder Project Files |
| 379 | Ticino-9 | SP |  |  | 0 | 0.530 |  |  |  |  | NA | Jefferies and Been (2006), Golder Project Files |
| 380 | Ticino Sand |  |  |  |  | 0.530 | 1.600 | 0.93 | 0.58 | 0.35 | NA | Borden (1991) |
| 381 | Ticino-8 | SP |  |  | 0 | 0.530 |  |  |  |  | NA | Jefferies and Been (2006), Golder Project Files |
| 382 | P3-S3 |  | 0.59 |  |  | 0.540 | 2.500 | 0.64 | 0.37 | 0.27 | ASTM D 4254 and ASTM D 4253 | Zheng and Hryciw (2016), Bareither et al. (2008) |
| 383 | Clean Sands (FC=0-5%) |  |  |  |  | 0.547 |  |  |  | 0.38 | Japanese Geotecnical Society-Procedures | Cubrinovski and Ishiara (2002) |
| 384 | Gravelly Sands (FC<6 %) |  |  |  |  | 0.547 |  |  |  | 0.32 | Japanese Geotecnical Society-Procedures | Cubrinovski and Ishiara (2002) |
| 385 | Niigata LD2-S7 |  |  |  |  | 0.550 | 2.700 | 0.99 | 0.55 | 0.44 | Japanese Standards | Ishiara et al (1978) |
| 386 | Niigata River Site D=8.5 m | SP |  |  | 1 | 0.550 | 2.700 | 0.99 | 0.55 | 0.44 | Japanese Society for Soil Mechanics and Foundation Engineering, 1979 | Skempton (1986) |
| 387 | Clean Sands (FC=0-5%) |  |  |  |  | 0.557 |  |  |  | 0.42 | Japanese Geotecnical Society-Procedures | Cubrinovski and Ishiara (2002) |
| 388 | Osterberg B1-S7/2 |  |  |  |  | 0.570 | 2.140 | 0.94 | 0.60 | 0.34 | Japanese Standards (Ishihara et al. 1978) | Ishiara et al (1979) |
| ***Data #*** | ***Soil Type*** | ***Soil Classification*** | ***R*** | ***S*** | ***FC*** | ***D_50_ (mm)*** | ***Cu*** | ***e_max_*** | ***e_min_*** | ***e_max_-e_min_*** | ***Test Methodology*** | ***Reference*** |
| 389 | Ottawa #45 Sand |  | 0.24 | 0.68 |  | 0.570 | 2.100 | 1.11 | 0.75 | 0.36 | NA | Zheng and Hryciw (2016), Sukumaran and Ashmawy (2001) |
| 390 | P4-S1 |  | 0.42 |  |  | 0.580 | 2.000 | 0.84 | 0.56 | 0.28 | ASTM D 4254 and ASTM D 4253 | Zheng and Hryciw (2016), Bareither et al. (2008) |
| 391 | Michigan 30A |  | 0.15 | 0.69 |  | 0.580 | 7.000 | 0.92 | 0.55 | 0.37 | NA | Zheng and Hryciw (2016) |
| 392 | Ticino Sand |  |  |  | 0 | 0.580 | 1.380 | 0.94 | 0.57 | 0.36 | NA | Santamarina and Cho (2001) |
| 393 | Ticino Sand |  | 0.4 | 0.8 |  | 0.580 | 1.500 | 0.99 | 0.57 | 0.42 | ASTM C136, D4254, and D1557 | Zheng and Hryciw (2016), Cho et al. (2006) |
| 394 | Daedalus Sand |  | 0.75 | 0.9 |  | 0.600 | 4.700 | 0.61 | 0.36 | 0.25 | NA | Zheng and Hryciw (2016), Thomann (1990) |
| 395 | ASTM 20/30 Sand |  | 0.8 | 0.9 |  | 0.600 | 1.400 | 0.69 |  |  | ASTM C136, D4254, and D1557 | Zheng and Hryciw (2016), Cho et al. (2006) |
| 396 | Hawaiian Sand |  | 0.6 | 0.75 |  | 0.600 | 1.500 | 0.86 | 0.52 | 0.34 | NA | Zheng and Hryciw (2016), Roberts (1964) |
| 397 | Brady, Texas |  | 0.68 | 0.76 |  | 0.610 | 1.400 | 0.84 | 0.57 | 0.27 | NA | Zheng and Hryciw (2016) |
| 398 | P3-S1 |  | 0.5 |  |  | 0.630 | 3.200 | 0.58 | 0.35 | 0.23 | ASTM D 4254 and ASTM D 4253 | Zheng and Hryciw (2016), Bareither et al. (2008) |
| 399 | Niigata River Site D=9.5 m/2 | SP |  |  | 2 | 0.630 | 2.800 | 0.99 | 0.51 | 0.48 | Japanese Society for Soil Mechanics and Foundation Engineering, 1979 | Skempton (1986) |
| 400 | Osterberg B3-S1 |  |  |  |  | 0.630 | 2.220 | 1.07 | 0.66 | 0.41 | Japanese Standards (Ishihara et al. 1978) | Ishiara et al (1979) |
| 401 | P2-S5 |  | 0.33 |  |  | 0.640 | 2.800 | 0.69 | 0.44 | 0.25 | ASTM D 4254 and ASTM D 4253 | Zheng and Hryciw (2016), Bareither et al. (2008) |
| 402 | Chesterton, Indiana Beach |  | 0.64 | 0.66 |  | 0.640 | 2.900 | 0.85 | 0.54 | 0.31 | NA | Zheng and Hryciw (2016) |
| 403 | Osterberg B1-S6/2 |  |  |  |  | 0.650 | 3.040 | 0.93 | 0.49 | 0.44 | Japanese Standards (Ishihara et al. 1978) | Ishiara et al (1979) |
| 404 | Niigata Road Site D=13.8 m/2 | SP |  |  |  | 0.650 | 1.900 | 1.05 | 0.56 | 0.49 | Japanese Society for Soil Mechanics and Foundation Engineering, 1979 | Skempton (1986) |
| 405 | Clean Sands (FC=0-5%) |  |  |  |  | 0.657 |  |  |  | 0.43 | Japanese Geotecnical Society-Procedures | Cubrinovski and Ishiara (2002) |
| 406 | Gravelly Sands (FC<6 %) |  |  |  |  | 0.657 |  |  |  | 0.41 | Japanese Geotecnical Society-Procedures | Cubrinovski and Ishiara (2002) |
| 407 | Niigata LD2-S10 |  |  |  |  | 0.680 | 3.000 | 1.02 | 0.54 | 0.48 | Japanese Standards | Ishiara et al (1978) |
| 408 | Gravelly Sands (FC<6 %) |  |  |  |  | 0.681 |  |  |  | 0.34 | Japanese Geotecnical Society-Procedures | Cubrinovski and Ishiara (2002) |
| 409 | P5-S1 |  | 0.38 |  |  | 0.690 | 5.300 | 0.55 | 0.31 | 0.24 | ASTM D 4254 and ASTM D 4253 | Zheng and Hryciw (2016), Bareither et al. (2008) |
| 410 | Cape Fear Sand |  |  |  |  | 0.690 | 2.760 | 0.80 | 0.53 | 0.28 | NA | Borden (1991) |
| 411 | P3-S4 |  | 0.52 |  |  | 0.700 | 2.900 | 0.60 | 0.37 | 0.23 | ASTM D 4254 and ASTM D 4253 | Zheng and Hryciw (2016), Bareither et al. (2008) |
| 412 | Small glass Beads |  | 1 | 1 |  | 0.700 | 1.100 | 0.75 | 0.50 | 0.25 | NA | Zheng and Hryciw (2016) |
| 413 | Niigata LD2-S8 |  |  |  |  | 0.700 | 3.000 | 1.00 | 0.48 | 0.52 | Japanese Standards | Ishiara et al (1978) |
| 414 | Niigata River Site D=9.5 m | SP |  |  | 3 | 0.700 | 3.000 | 1.00 | 0.48 | 0.52 | Japanese Society for Soil Mechanics and Foundation Engineering, 1979 | Skempton (1986) |
| 415 | Ottawa 20–30 |  | 0.75 | 0.82 |  | 0.710 | 1.400 | 0.74 | 0.49 | 0.25 | NA | Zheng and Hryciw (2016) |
| 416 | Blasting Sand |  |  |  | 0 | 0.710 | 1.940 | 1.03 | 0.70 | 0.33 | NA | Santamarina and Cho (2001) |
| ***Data #*** | ***Soil Type*** | ***Soil Classification*** | ***R*** | ***S*** | ***FC*** | ***D_50_ (mm)*** | ***Cu*** | ***e_max_*** | ***e_min_*** | ***e_max_-e_min_*** | ***Test Methodology*** | ***Reference*** |
| 417 | Blasting Sand |  | 0.3 | 0.55 |  | 0.710 | 1.900 | 1.03 | 0.70 | 0.33 | ASTM C136, D4254, and D1557 | Zheng and Hryciw (2016), Cho et al. (2006) |
| 418 | Gravelly Sands (FC<6 %) |  |  |  |  | 0.720 |  |  |  | 0.35 | Japanese Geotecnical Society-Procedures | Cubrinovski and Ishiara (2002) |
| 419 | Trakya |  | 0.35 | 0.65 |  | 0.720 | 6.300 | 0.70 | 0.49 | 0.21 | ASTM D4253 and D4254 | Zheng and Hryciw (2016), Cabalar et al. (2013) |
| 420 | Ottawa #20/30 Sand |  | 0.9 | 0.9 |  | 0.720 | 1.200 | 0.74 | 0.50 | 0.24 | ASTM C136, D4254, and D1557 | Zheng and Hryciw (2016), Cho et al. (2006) |
| 421 | Ottawa Sand |  | 0.8 | 0.9 |  | 0.720 | 1.400 | 0.74 | 0.50 | 0.24 | NA | Zheng and Hryciw (2016), Georgiannou and Konstadinou (2013) |
| 422 | Ottawa 20–30 |  |  |  | 0 | 0.720 | 1.150 | 0.74 | 0.50 | 0.24 | NA | Santamarina and Cho (2001) |
| 423 | Ottawa 20–30 |  | 0.65 | 0.87 |  | 0.720 | 1.200 | 0.78 | 0.46 | 0.32 | NA | Zheng and Hryciw (2016), Zelasko et al. (1975) |
| 424 | Evanston Beach 20–30 |  | 0.44 | 0.71 |  | 0.720 | 1.200 | 0.92 | 0.55 | 0.37 | NA | Zheng and Hryciw (2016), Zelasko et al. (1975) |
| 425 | Franklin Falls 20–30 |  | 0.36 | 0.52 |  | 0.720 | 1.200 | 1.08 | 0.62 | 0.46 | NA | Zheng and Hryciw (2016), Zelasko et al. (1975) |
| 426 | West Kowloon Sand | SP |  |  | 0.5 | 0.730 |  | 0.69 | 0.44 | 0.24 | NA | Jefferies and Been (2006), Golder Project Files |
| 427 | Gravelly Sands (FC<6 %) |  |  |  |  | 0.733 |  |  |  | 0.32 | Japanese Geotecnical Society-Procedures | Cubrinovski and Ishiara (2002) |
| 428 | Sands with Fines (5<FC<15%) |  |  |  |  | 0.733 |  |  |  | 0.44 | Japanese Geotecnical Society-Procedures | Cubrinovski and Ishiara (2002) |
| 429 | Ottawa 20–30 |  | 0.78 | 0.9 |  | 0.740 | 1.100 | 0.74 | 0.51 | 0.23 | ASTM D4254-91, Method B and ASTM D4253-93, Method 2A | Zheng and Hryciw (2016), DeJong and Christoph (2009) |
| 430 | Griffin, Indiana |  | 0.6 | 0.69 |  | 0.740 | 4.300 | 0.79 | 0.51 | 0.28 | NA | Zheng and Hryciw (2016) |
| 431 | Ottawa 20–30 |  | 0.75 | 0.9 |  | 0.750 | 1.200 | 0.72 | 0.51 | 0.21 | NA | Zheng and Hryciw (2016), Thomann (1990) |
| 432 | Clean Sand |  | 0.4 | 0.68 |  | 0.750 | 3.800 | 0.90 | 0.48 | 0.42 | NA | Zheng and Hryciw (2016), Kumar and Madhusudhan (2012) |
| 433 | Q-Rok |  | 0.2 | 0.5 |  | 0.750 | 1.500 | 1.14 | 0.70 | 0.44 | ASTM D4254-91, Method B and ASTM D4253-93, Method 2A | Zheng and Hryciw (2016), DeJong and Christoph (2009) |
| 434 | Ottowa Sand | SP |  |  |  | 0.753 | 1.400 | 0.69 | 0.39 | 0.30 | ASTM D 2049-69 | Youd (1973) |
| 435 | Monterey Sand | SP |  |  |  | 0.753 | 1.400 | 0.77 | 0.44 | 0.33 | ASTM D 2049-69 | Youd (1973) |
| 436 | Crushed Basalt |  |  |  |  | 0.753 | 1.400 | 1.24 | 0.69 | 0.55 | ASTM D 2049-69 | Youd (1973) |
| 437 | Abraded Leighton Buzzard |  | 0.75 | 0.8 |  | 0.760 | 1.300 | 0.80 | 0.51 | 0.29 | NA | Zheng and Hryciw (2016), Lings and Dietz (2004) |
| 438 | P4-S3 |  | 0.35 |  |  | 0.770 | 6.500 | 0.62 | 0.33 | 0.29 | ASTM D 4254 and ASTM D 4253 | Zheng and Hryciw (2016), Bareither et al. (2008) |
| 439 | Bushehr Port |  | 0.35 | 0.6 |  | 0.780 | 4.500 | 0.91 | 0.63 | 0.28 | ASTM | Zheng and Hryciw (2016), Shahnazari and Rezvai (2013) |
| 440 | Crushed gabbro |  | 0.23 | 0.56 |  | 0.800 | 5.500 | 0.96 | 0.60 | 0.36 | NA | Zheng and Hryciw (2016) |
| 441 | Clean Sands (FC=0-5%) |  |  |  |  | 0.818 |  |  |  | 0.25 | Japanese Geotecnical Society-Procedures | Cubrinovski and Ishiara (2002) |
| 442 | Gravelly Sands (FC<6 %) |  |  |  |  | 0.848 |  |  |  | 0.36 | Japanese Geotecnical Society-Procedures | Cubrinovski and Ishiara (2002) |
| 443 | Gravelly Sands (FC<6 %) |  |  |  |  | 0.848 |  |  |  | 0.29 | Japanese Geotecnical Society-Procedures | Cubrinovski and Ishiara (2002) |
| 444 | Leighton Buzzard |  |  |  |  | 0.850 | 1.300 | 0.79 | 0.49 | 0.30 | NA | Mayne and Kulhawy (1991), Houlsby and Hitchman (1988) |
| 445 | Birecik |  | 0.65 | 0.72 |  | 0.860 | 3.300 | 0.80 | 0.55 | 0.25 | ASTM D4253 and D4254 | Zheng and Hryciw (2016), Cabalar et al. (2013) |
| 446 | Leighton Buzzard |  | 0.5 | 0.82 |  | 0.860 | 1.200 | 0.82 | 0.54 | 0.28 | NA | Zheng and Hryciw (2016), Sladen et al. (1985) |
| 447 | Gravelly Sands (FC<6 %) |  |  |  |  | 0.913 |  |  |  | 0.28 | Japanese Geotecnical Society-Procedures | Cubrinovski and Ishiara (2002) |
| ***Data #*** | ***Soil Type*** | ***Soil Classification*** | ***R*** | ***S*** | ***FC*** | ***D_50_ (mm)*** | ***Cu*** | ***e_max_*** | ***e_min_*** | ***e_max_-e_min_*** | ***Test Methodology*** | ***Reference*** |
| 448 | Gravelly Sands (FC<6 %) |  |  |  |  | 0.947 |  |  |  | 0.31 | Japanese Geotecnical Society-Procedures | Cubrinovski and Ishiara (2002) |
| 449 | Gravelly Sands (FC<6 %) |  |  |  |  | 0.964 |  |  |  | 0.35 | Japanese Geotecnical Society-Procedures | Cubrinovski and Ishiara (2002) |
| 450 | Large glass Beads |  | 1 | 1 |  | 0.970 | 1.100 | 0.74 | 0.50 | 0.24 | NA | Zheng and Hryciw (2016) |
| 451 | Chek Lap Kok | SP |  |  | 0.5 | 1.000 |  | 0.68 | 0.41 | 0.27 | NA | Jefferies and Been (2006), Golder Project Files |
| 452 | Lone Star 2 |  |  |  |  | 1.000 | 2.000 | 0.77 | 0.48 | 0.28 | NA | Mayne and Kulhawy (1991), Villet and Mitchell (1981) |
| 453 | Narli |  | 0.75 | 0.65 |  | 1.000 | 3.700 | 0.83 | 0.52 | 0.31 | ASTM D4253 and D4254 | Zheng and Hryciw (2016), Cabalar et al. (2013) |
| 454 | Gravels |  |  |  |  | 1.056 |  |  |  | 0.28 | Japanese Geotecnical Society-Procedures | Cubrinovski and Ishiara (2002) |
| 455 | G25 Gravel | SP |  |  |  | 1.130 | 5.650 | 0.57 | 0.33 | 0.23 | ASTM | Kokusho and Yoshida (1997) |
| 456 | Crushed stone Sand |  | 0.45 | 0.61 |  | 1.400 | 2.500 | 0.93 | 0.62 | 0.31 | ASTM D4253 and D4254 | Zheng and Hryciw (2016), Cabalar et al. (2013) |
| 457 | Monterey Sand | SP |  |  |  | 1.497 | 1.400 | 0.75 | 0.45 | 0.30 | ASTM D 2049-69 | Youd (1973) |
| 458 | Cambria Sand | SP |  |  | 0 | 1.500 | 1.304 | 0.77 | 0.54 | 0.23 | Non-Standard Procedures | Cubrinovski and Ishiara (2002), Lade et al. (1998) |
| 459 | Long-grain rice |  | 0.62 | 0.4 |  | 1.510 | 1.100 | 1.08 | 0.85 | 0.23 | NA | Zheng and Hryciw (2016) |
| 460 | Crushed Basalt |  |  |  |  | 1.520 | 1.400 | 1.23 | 0.70 | 0.53 | ASTM D 2049-69 | Youd (1973) |
| 461 | Sand L |  | 0.14 | 0.57 |  | 1.640 | 2.000 | 1.20 | 0.62 | 0.58 | ASTM D4253-93 and ASTM D4254-91 | Zheng and Hryciw (2016), Guo and Su (2007) |
| 462 | Fused aluminum oxide |  | 0.3 | 0.69 |  | 1.800 | 1.600 | 0.92 | 0.63 | 0.29 | NA | Zheng and Hryciw (2016) |
| 463 | Short-grain rice |  | 0.54 | 0.55 |  | 1.910 | 1.100 | 0.97 | 0.65 | 0.32 | NA | Zheng and Hryciw (2016) |
| 464 | Gravels |  |  |  |  | 2.154 |  |  |  | 0.21 | Japanese Geotecnical Society-Procedures | Cubrinovski and Ishiara (2002) |
| 465 | G50 Gravel | GW |  |  |  | 2.280 | 11.300 | 0.43 | 0.24 | 0.19 | ASTM | Kokusho and Yoshida (1997) |
| 466 | Lapis Lustre Sand |  |  |  |  | 3.024 | 1.400 | 0.73 | 0.44 | 0.29 | ASTM D 2049-69 | Youd (1973) |
| 467 | Crushed Basalt |  |  |  |  | 3.024 | 1.400 | 1.16 | 0.68 | 0.48 | ASTM D 2049-69 | Youd (1973) |
| 468 | Gravels |  |  |  |  | 3.465 |  |  |  | 0.27 | Japanese Geotecnical Society-Procedures | Cubrinovski and Ishiara (2002) |
| 469 | Gravels |  |  |  |  | 3.465 |  |  |  | 0.21 | Japanese Geotecnical Society-Procedures | Cubrinovski and Ishiara (2002) |
| 470 | P2-S11 |  | 0.52 |  |  | 3.500 | 3.400 | 0.43 | 0.26 | 0.17 | ASTM D 4254 and ASTM D 4253 | Zheng and Hryciw (2016), Bareither et al. (2008) |
| 471 | Gravels |  |  |  |  | 3.529 |  |  |  | 0.24 | Japanese Geotecnical Society-Procedures | Cubrinovski and Ishiara (2002) |
| 472 | Gravels |  |  |  |  | 4.903 |  |  |  | 0.23 | Japanese Geotecnical Society-Procedures | Cubrinovski and Ishiara (2002) |
| 473 | Gravels |  |  |  |  | 5.886 |  |  |  | 0.21 | Japanese Geotecnical Society-Procedures | Cubrinovski and Ishiara (2002) |
| 474 | Gravels |  |  |  |  | 5.995 |  |  |  | 0.25 | Japanese Geotecnical Society-Procedures | Cubrinovski and Ishiara (2002) |
| 475 | Gravels |  |  |  |  | 6.449 |  |  |  | 0.22 | Japanese Geotecnical Society-Procedures | Cubrinovski and Ishiara (2002) |
| 476 | Gravels |  |  |  |  | 7.067 |  |  |  | 0.19 | Japanese Geotecnical Society-Procedures | Cubrinovski and Ishiara (2002) |
| 477 | Gravels |  |  |  |  | 7.197 |  |  |  | 0.23 | Japanese Geotecnical Society-Procedures | Cubrinovski and Ishiara (2002) |
| 478 | G75 Gravel | GW |  |  |  | 7.300 | 31.100 | 0.35 | 0.18 | 0.17 | ASTM | Kokusho and Yoshida (1997) |
| 479 | Gravels |  |  |  |  | 7.602 |  |  |  | 0.27 | Japanese Geotecnical Society-Procedures | Cubrinovski and Ishiara (2002) |
| ***Data #*** | ***Soil Type*** | ***Soil Classification*** | ***R*** | ***S*** | ***FC*** | ***D_50_ (mm)*** | ***Cu*** | ***e_max_*** | ***e_min_*** | ***e_max_-e_min_*** | ***Test Methodology*** | ***Reference*** |
| 480 | Gravels |  |  |  |  | 8.031 |  |  |  | 0.23 | Japanese Geotecnical Society-Procedures | Cubrinovski and Ishiara (2002) |
| 481 | Gravels |  |  |  |  | 8.483 |  |  |  | 0.23 | Japanese Geotecnical Society-Procedures | Cubrinovski and Ishiara (2002) |
| 482 | Gravels |  |  |  |  | 9.127 |  |  |  | 0.26 | Japanese Geotecnical Society-Procedures | Cubrinovski and Ishiara (2002) |
| 483 | Gravels |  |  |  |  | 9.127 |  |  |  | 0.23 | Japanese Geotecnical Society-Procedures | Cubrinovski and Ishiara (2002) |
| 484 | Gravels |  |  |  |  | 10.184 |  |  |  | 0.27 | Japanese Geotecnical Society-Procedures | Cubrinovski and Ishiara (2002) |
| 485 | Gravels |  |  |  |  | 10.564 |  |  |  | 0.19 | Japanese Geotecnical Society-Procedures | Cubrinovski and Ishiara (2002) |
| 486 | Gravels |  |  |  |  | 12.226 |  |  |  | 0.19 | Japanese Geotecnical Society-Procedures | Cubrinovski and Ishiara (2002) |
| 487 | Gravels |  |  |  |  | 12.452 |  |  |  | 0.25 | Japanese Geotecnical Society-Procedures | Cubrinovski and Ishiara (2002) |
| 488 | Gravels |  |  |  |  | 12.452 |  |  |  | 0.26 | Japanese Geotecnical Society-Procedures | Cubrinovski and Ishiara (2002) |
| 489 | Gravels |  |  |  |  | 13.396 |  |  |  | 0.29 | Japanese Geotecnical Society-Procedures | Cubrinovski and Ishiara (2002) |
| 490 | Gravels |  |  |  |  | 14.678 |  |  |  | 0.24 | Japanese Geotecnical Society-Procedures | Cubrinovski and Ishiara (2002) |
| 491 | Gravels |  |  |  |  | 16.379 |  |  |  | 0.27 | Japanese Geotecnical Society-Procedures | Cubrinovski and Ishiara (2002) |
| 492 | Gravels |  |  |  |  | 18.957 |  |  |  | 0.27 | Japanese Geotecnical Society-Procedures | Cubrinovski and Ishiara (2002) |
| 493 | Mix4 |  | 0.37 |  |  | 0.740 | 8.000 | 0.49 | 0.27 | 0.22 | ASTM D 2049-69 | Youd (1973) |
| 494 | Mix3 |  | 0.37 |  |  | 0.740 | 4.300 | 0.58 | 0.30 | 0.28 | ASTM D 2049-69 | Youd (1973) |
| 495 | Sılt |  |  |  | 20 |  |  | 0.62 | 0.29 | 0.33 | ASTM D 4253 and ASTM D 4254 | Salgado et al. (2000) |
| 496 | Sılt |  |  |  | 15 |  |  | 0.63 | 0.32 | 0.31 | ASTM D 4253 and ASTM D 4254 | Salgado et al. (2000) |
| 497 | Mix5 |  | 0.37 |  |  | 0.740 | 4.300 | 0.64 | 0.34 | 0.31 | ASTM D 2049-69 | Youd (1973) |
| 498 | Sılt |  |  |  | 10 |  |  | 0.65 | 0.36 | 0.29 | ASTM D 4253 and ASTM D 4254 | Salgado et al. (2000) |
| 499 | Mix2 |  | 0.35 |  |  | 0.740 | 2.500 | 0.69 | 0.37 | 0.32 | ASTM D 2049-69 | Youd (1973) |
| 500 | Badger Sand |  | 0.81 | 0.9 |  |  | 1.300 | 0.69 | 0.49 | 0.20 | ASTM D 4253 (ASTM, 2006a) and ASTM D 4254 Method B (ASTM, 2006b) | Zheng and Hryciw (2016), Rousé et al. (2008) |
| 501 | Sılt |  |  |  | 5 |  |  | 0.70 | 0.42 | 0.28 | ASTM D 4253 and ASTM D 4254 | Salgado et al. (2000) |
| 502 | Beach gravel |  | 0.58 | 0.74 |  |  | 1.600 | 0.74 | 0.53 | 0.21 | NA | Zheng and Hryciw (2016), Morroto and Ishii (1990) |
| 503 | Bronze ballotini |  | 0.8 | 0.8 |  |  | 1.600 | 0.74 | 0.54 | 0.20 | NA | Zheng and Hryciw (2016), Barden et al. (1969) |
| 504 | Berlin |  |  |  |  |  | 2.273 | 0.75 | 0.46 | 0.29 | NA | Bolton (1986), DeBeer (1965) |
| 505 | Sılt |  |  |  | 0 |  |  | 0.78 | 0.48 | 0.30 | ASTM D 4253 and ASTM D 4254 | Salgado et al. (2000) |
| 506 | Ottawa Sand (round) |  |  |  |  |  | 1.480 | 0.78 | 0.48 | 0.30 | ASTM C778 | Salgado et al. (2000) |
| 507 | Monterey no 20 |  |  |  |  |  | 2.000 | 0.78 | 0.57 | 0.21 | NA | Bolton (1986), Marachi, Chan, Seed and Duncan (1969) |
| ***Data #*** | ***Soil Type*** | ***Soil Classification*** | ***R*** | ***S*** | ***FC*** | ***D_50_ (mm)*** | ***Cu*** | ***e_max_*** | ***e_min_*** | ***e_max_-e_min_*** | ***Test Methodology*** | ***Reference*** |
| 508 | Brasted River |  |  |  |  |  | 2.417 | 0.79 | 0.47 | 0.32 | Procedure proposed by Kolbuszewski (1948) | Bolton (1986), Cornforth (1964, 1973) |
| 509 | Leighton Buzzard 14/25 |  |  |  |  |  | 1.308 | 0.79 | 0.49 | 0.30 | NA | Bolton (1986), Stroud (1971) |
| 510 | Mix1 |  | 0.34 |  |  | 0.740 | 1.400 | 0.80 | 0.46 | 0.34 | ASTM D 2049-69 | Youd (1973) |
| 511 | CB-4 |  | 0.19 |  |  | 0.740 | 8.000 | 0.80 | 0.44 | 0.36 | ASTM D 2049-69 | Youd (1973) |
| 512 | Ottawa Sand |  |  |  |  |  | 1.169 | 0.80 | 0.49 | 0.31 | NA | Bolton (1986), Lee and Seed (1967) |
| 513 | Mersey River |  |  |  |  |  | 2.000 | 0.82 | 0.49 | 0.33 | NA | Bolton (1986), Rowe (1969) & Rowe and Barden (1964) |
| 514 | Karlsruhe medium Sand |  |  |  |  |  | 2.900 | 0.82 | 0.54 | 0.28 | NA | Bolton (1986), Hettler (1981) |
| 515 | River gravel |  | 0.43 | 0.75 |  |  | 1.600 | 0.84 | 0.55 | 0.29 | NA | Zheng and Hryciw (2016), Morroto and Ishii (1990) |
| 516 | Glacial outwash Sand |  |  |  |  |  | 6.000 | 0.84 | 0.41 | 0.43 | ASTM | Bolton (1986), Hirscfield and Poulos (1964) |
| 517 | Monterey #0 Sand |  |  |  |  |  | 1.600 | 0.86 | 0.57 | 0.29 | ASTM | Salgado et al. (2000) , Bolton (1986) & Chung et al. (1984) |
| 518 | Monterey no 0 |  |  |  |  |  | 1.667 | 0.86 | 0.57 | 0.29 | NA | Bolton (1986), Lade and Duncan (1973) |
| 519 | Hokksund Sand |  |  |  |  |  | 1.910 | 0.87 | 0.55 | 0.32 | ASTM | Salgado et al. (2000) , Lo Presti (1987) & Lo Presti et al. (1992) |
| 520 | Drammen |  |  |  |  |  | 2.700 | 0.89 | 0.43 | 0.46 | NA | Lunne (1991) |
| 521 | Ticino |  |  |  |  |  | 1.130 | 0.89 | 0.52 | 0.37 | NA | Lunne (1991) |
| 522 | Mol |  |  |  |  |  | 1.500 | 0.89 | 0.56 | 0.33 | NA | Bolton (1986), Ladanyi (1960) |
| 523 | Crushed feldspar |  | 0.25 | 0.65 |  |  | 6.400 | 0.90 | 0.56 | 0.34 | NA | Zheng and Hryciw (2016), Barden et al. (1969) |
| 524 | Gabbro |  | 0.27 | 0.74 |  |  | 1.600 | 0.90 | 0.65 | 0.25 | NA | Zheng and Hryciw (2016), Morroto and Ishii (1990) |
| 525 | Guinea Marine |  |  |  |  |  | 2.563 | 0.90 | 0.52 | 0.38 | Procedure proposed by Kolbuszewski (1948) | Bolton (1986), Cornforth (1973) |
| 526 | Hokksund |  |  |  |  |  | 1.850 | 0.92 | 0.56 | 0.36 | NA | Lunne (1991) |
| 527 | Ham River |  |  |  |  |  | 1.563 | 0.92 | 0.59 | 0.33 | Procedure proposed by Kolbuszewski (1948) | Bolton (1986), Bishop and Green (1965) |
| 528 | Ticino Sand |  |  |  |  |  | 1.500 | 0.93 | 0.57 | 0.36 | ASTM | Salgado et al. (2000) , Lo Presti (1987) & Lo Presti et al. (1992) |
| 529 | River Welland Sand |  | 0.4 | 0.6 |  |  | 1.700 | 0.94 | 0.62 | 0.32 | NA | Zheng and Hryciw (2016), Barden et al. (1969) |
| 530 | Welland River |  |  |  |  |  | 1.400 | 0.94 | 0.62 | 0.32 | NA | Bolton (1986), Barden et al. (1969) |
| 531 | Dolelite |  | 0.39 | 0.76 |  |  | 1.600 | 0.96 | 0.69 | 0.27 | NA | Zheng and Hryciw (2016), Morroto and Ishii (1990) |
| 532 | SP | SP |  |  |  |  |  | 0.96 | 0.60 | 0.36 | Japanese Geotecnical Society-Procedures | Cubrinovski and Ishiara (2002) |
| 533 | Greywacke |  | 0.31 | 0.65 |  |  | 1.600 | 0.97 | 0.72 | 0.25 | NA | Zheng and Hryciw (2016), Morroto and Ishii (1990) |
| 534 | SP | SP |  |  |  |  |  | 0.98 | 0.61 | 0.37 | Japanese Geotecnical Society-Procedures | Cubrinovski and Ishiara (2002) |
| 535 | Toyoura Sand |  |  |  |  |  | 1.270 | 0.99 | 0.61 | 0.38 | ASTM | Salgado et al. (2000) , Lo Presti (1987) & Lo Presti et al. (1992) |
| 536 | Onahama |  | 0.32 | 0.72 |  |  | 1.600 | 0.99 | 0.71 | 0.28 | NA | Zheng and Hryciw (2016), Morroto and Ishii (1990) |
| 537 | CB-3 |  | 0.19 |  |  | 0.740 | 4.300 | 0.99 | 0.48 | 0.51 | ASTM D 2049-69 | Youd (1973) |
| 538 | Sleipner |  |  |  |  |  | 1.560 | 1.00 | 0.54 | 0.46 | NA | Lunne (1991), Lunne (1991) |
| 539 | Sacramento River Sand | SP |  |  |  |  | 1.470 | 1.03 | 0.61 | 0.42 | NA | Bolton (1986), Lee & Seed (1967) |
| 540 | CB-2 |  | 0.19 |  |  | 0.740 | 2.500 | 1.10 | 0.59 | 0.51 | ASTM D 2049-69 | Youd (1973) |
| ***Data #*** | ***Soil Type*** | ***Soil Classification*** | ***R*** | ***S*** | ***FC*** | ***D_50_ (mm)*** | ***Cu*** | ***e_max_*** | ***e_min_*** | ***e_max_-e_min_*** | ***Test Methodology*** | ***Reference*** |
| 541 | Chattahoochee River |  |  |  |  |  | 2.238 | 1.10 | 0.61 | 0.49 | NA | Bolton (1986), Vesic and Clough (1968) |
| 542 | Portland River |  |  |  |  |  | 1.565 | 1.10 | 0.63 | 0.47 | Procedure proposed by Kolbuszewski (1948) | Bolton (1986), Cornforth (1973) |
| 543 | Limassol Marine |  |  |  |  |  | 36.667 | 1.18 | 0.57 | 0.61 | Procedure proposed by Kolbuszewski (1948) | Bolton (1986), Cornforth (1973) |
| 544 | CB-1 |  | 0.19 |  |  | 0.740 | 1.400 | 1.26 | 0.71 | 0.55 | ASTM D 2049-69 | Youd (1973) |
| 545 | Aged Sand |  |  |  |  | 0.089 | 1.511 | 1.05 | 0.63 | 0.43 | ASTM D4253 and D4254 | Chen and Kulhawy (2014) |
| 546 | Aged Sand |  |  |  |  | 0.114 | 2.007 | 1.10 | 0.63 | 0.47 | ASTM D4253 and D4254 | Chen and Kulhawy (2014) |
| 547 | Aged Sand |  |  |  |  | 0.128 | 2.119 | 1.03 | 0.60 | 0.43 | ASTM D4253 and D4254 | Chen and Kulhawy (2014) |
| 548 | Aged Sand |  |  |  |  | 0.147 | 2.330 | 0.99 | 0.61 | 0.38 | ASTM D4253 and D4254 | Chen and Kulhawy (2014) |
| 549 | Quaternary Sand |  |  |  |  | 0.252 | 1.393 | 1.05 | 0.67 | 0.38 | ASTM D4253 and D4254 | Chen and Kulhawy (2014) |
| 550 | Quaternary Sand |  |  |  |  | 0.290 | 1.491 | 1.08 | 0.64 | 0.45 | ASTM D4253 and D4254 | Chen and Kulhawy (2014) |
| 551 | Quaternary Sand |  |  |  |  | 0.200 | 1.563 | 1.06 | 0.70 | 0.36 | ASTM D4253 and D4254 | Chen and Kulhawy (2014) |
| 552 | Quaternary Sand |  |  |  |  | 0.230 | 1.595 | 0.91 | 0.62 | 0.29 | ASTM D4253 and D4254 | Chen and Kulhawy (2014) |
| 553 | Quaternary Sand |  |  |  |  | 0.161 | 1.695 | 1.15 | 0.65 | 0.49 | ASTM D4253 and D4254 | Chen and Kulhawy (2014) |
| 554 | Quaternary Sand |  |  |  |  | 0.290 | 1.777 | 1.10 | 0.70 | 0.40 | ASTM D4253 and D4254 | Chen and Kulhawy (2014) |
| 555 | Quaternary Sand |  |  |  |  | 0.160 | 1.789 | 1.21 | 0.76 | 0.45 | ASTM D4253 and D4254 | Chen and Kulhawy (2014) |
| 556 | Quaternary Sand |  |  |  |  | 0.457 | 1.801 | 1.03 | 0.68 | 0.35 | ASTM D4253 and D4254 | Chen and Kulhawy (2014) |
| 557 | Quaternary Sand |  |  |  |  | 0.202 | 1.801 | 1.13 | 0.56 | 0.57 | ASTM D4253 and D4254 | Chen and Kulhawy (2014) |
| 558 | Quaternary Sand |  |  |  |  | 0.197 | 2.007 | 0.84 | 0.57 | 0.27 | ASTM D4253 and D4254 | Chen and Kulhawy (2014) |
| 559 | Quaternary Sand |  |  |  |  | 0.460 | 2.007 | 1.02 | 0.50 | 0.52 | ASTM D4253 and D4254 | Chen and Kulhawy (2014) |
| 560 | Quaternary Sand |  |  |  |  | 0.562 | 2.077 | 1.19 | 0.78 | 0.41 | ASTM D4253 and D4254 | Chen and Kulhawy (2014) |
| 561 | Quaternary Sand |  |  |  |  | 0.161 | 2.177 | 0.94 | 0.65 | 0.29 | ASTM D4253 and D4254 | Chen and Kulhawy (2014) |
| 562 | Quaternary Sand |  |  |  |  | 0.181 | 2.237 | 1.13 | 0.75 | 0.38 | ASTM D4253 and D4254 | Chen and Kulhawy (2014) |
| 563 | Quaternary Sand |  |  |  |  | 0.200 | 2.394 | 0.94 | 0.65 | 0.29 | ASTM D4253 and D4254 | Chen and Kulhawy (2014) |
| 564 | Quaternary Sand |  |  |  |  | 0.277 | 2.410 | 1.11 | 0.53 | 0.58 | ASTM D4253 and D4254 | Chen and Kulhawy (2014) |
| 565 | Quaternary Sand |  |  |  |  | 0.301 | 2.493 | 1.15 | 0.76 | 0.39 | ASTM D4253 and D4254 | Chen and Kulhawy (2014) |
| 566 | Quaternary Sand |  |  |  |  | 0.631 | 2.797 | 0.99 | 0.51 | 0.48 | ASTM D4253 and D4254 | Chen and Kulhawy (2014) |
| 567 | Sand Fill |  |  |  |  | 0.250 | 1.201 | 0.84 | 0.52 | 0.32 | ASTM D4253 and D4254 | Chen and Kulhawy (2014) |
| 568 | Sand Fill |  |  |  |  | 0.323 | 1.685 | 1.00 | 0.63 | 0.38 | ASTM D4253 and D4254 | Chen and Kulhawy (2014) |
| 569 | Sand Fill |  |  |  |  | 0.210 | 1.995 | 1.41 | 0.90 | 0.52 | ASTM D4253 and D4254 | Chen and Kulhawy (2014) |
| 570 | Sand Fill |  |  |  |  | 0.211 | 2.077 | 1.15 | 0.72 | 0.43 | ASTM D4253 and D4254 | Chen and Kulhawy (2014) |
| 571 | Sand Fill |  |  |  |  | 0.232 | 2.106 | 1.22 | 0.72 | 0.49 | ASTM D4253 and D4254 | Chen and Kulhawy (2014) |
| 572 | Sand Fill |  |  |  |  | 0.301 | 4.008 | 1.08 | 0.57 | 0.51 | ASTM D4253 and D4254 | Chen and Kulhawy (2014) |
| 573 | Tailing Sand |  |  |  |  | 0.159 | 2.207 | 0.96 | 0.52 | 0.44 | ASTM D4253 and D4254 | Chen and Kulhawy (2014) |
| 574 | Tailing Sand |  |  |  |  | 0.170 | 2.476 | 0.99 | 0.46 | 0.53 | ASTM D4253 and D4254 | Chen and Kulhawy (2014) |
| 575 | Tailing Sand |  |  |  |  | 0.200 | 2.759 | 1.06 | 0.54 | 0.51 | ASTM D4253 and D4254 | Chen and Kulhawy (2014) |
| 576 | Tailing Sand |  |  |  |  | 0.252 | 3.949 | 1.01 | 0.51 | 0.51 | ASTM D4253 and D4254 | Chen and Kulhawy (2014) |
| 577 | Volcanic Sand |  |  |  |  | 0.533 | 3.496 | 1.71 | 1.08 | 0.63 | ASTM D4253 and D4254 | Chen and Kulhawy (2014) |
| 578 | Volcanic Sand |  |  |  |  | 0.380 | 10.969 | 2.38 | 1.45 | 0.93 | ASTM D4253 and D4254 | Chen and Kulhawy (2014) |
| 579 | Volcanic Sand |  |  |  |  | 0.453 | 11.896 | 1.71 | 0.96 | 0.75 | ASTM D4253 and D4254 | Chen and Kulhawy (2014) |
| 580 | Volcanic Sand |  |  |  |  | 0.371 | 14.475 | 1.59 | 0.96 | 0.64 | ASTM D4253 and D4254 | Chen and Kulhawy (2014) |
| 581 | Quaternary Gravel |  |  |  |  | 0.318 | 3.227 | 0.62 | 0.35 | 0.28 | ASTM D4253 and D4254 | Chen and Kulhawy (2014) |
| ***Data #*** | ***Soil Type*** | ***Soil Classification*** | ***R*** | ***S*** | ***FC*** | ***D_50_ (mm)*** | ***Cu*** | ***e_max_*** | ***e_min_*** | ***e_max_-e_min_*** | ***Test Methodology*** | ***Reference*** |
| 582 | Quaternary Gravel |  |  |  |  | 1.668 | 5.080 | 0.54 | 0.28 | 0.26 | ASTM D4253 and D4254 | Chen and Kulhawy (2014) |
| 583 | Quaternary Gravel |  |  |  |  | 1.206 | 5.739 | 0.68 | 0.37 | 0.32 | ASTM D4253 and D4254 | Chen and Kulhawy (2014) |
| 584 | Quaternary Gravel |  |  |  |  | 1.801 | 8.616 | 0.62 | 0.31 | 0.31 | ASTM D4253 and D4254 | Chen and Kulhawy (2014) |
| 585 | Quaternary Gravel |  |  |  |  | 2.839 | 10.344 | 0.63 | 0.35 | 0.29 | ASTM D4253 and D4254 | Chen and Kulhawy (2014) |
| 586 | Quaternary Gravel |  |  |  |  | 4.508 | 12.420 | 0.53 | 0.29 | 0.24 | ASTM D4253 and D4254 | Chen and Kulhawy (2014) |
| 587 | Quaternary Gravel |  |  |  |  | 10.287 | 14.030 | 0.41 | 0.20 | 0.21 | ASTM D4253 and D4254 | Chen and Kulhawy (2014) |
| 588 | Quaternary Gravel |  |  |  |  | 24.964 | 14.030 | 0.70 | 0.42 | 0.28 | ASTM D4253 and D4254 | Chen and Kulhawy (2014) |
| 589 | Quaternary Gravel |  |  |  |  | 24.964 | 14.221 | 0.55 | 0.35 | 0.20 | ASTM D4253 and D4254 | Chen and Kulhawy (2014) |
| 590 | Quaternary Gravel |  |  |  |  | 7.050 | 15.013 | 0.59 | 0.43 | 0.17 | ASTM D4253 and D4254 | Chen and Kulhawy (2014) |
| 591 | Quaternary Gravel |  |  |  |  | 8.817 | 15.013 | 0.67 | 0.39 | 0.28 | ASTM D4253 and D4254 | Chen and Kulhawy (2014) |
| 592 | Quaternary Gravel |  |  |  |  | 13.788 | 17.425 | 0.59 | 0.38 | 0.21 | ASTM D4253 and D4254 | Chen and Kulhawy (2014) |
| 593 | Quaternary Gravel |  |  |  |  | 4.272 | 19.818 | 0.60 | 0.40 | 0.20 | ASTM D4253 and D4254 | Chen and Kulhawy (2014) |
| 594 | Quaternary Gravel |  |  |  |  | 3.261 | 20.362 | 0.48 | 0.22 | 0.26 | ASTM D4253 and D4254 | Chen and Kulhawy (2014) |
| 595 | Quaternary Gravel |  |  |  |  | 10.052 | 20.780 | 0.58 | 0.33 | 0.25 | ASTM D4253 and D4254 | Chen and Kulhawy (2014) |
| 596 | Quaternary Gravel |  |  |  |  | 10.609 | 20.921 | 0.61 | 0.44 | 0.16 | ASTM D4253 and D4254 | Chen and Kulhawy (2014) |
| 597 | Quaternary Gravel |  |  |  |  | 2.928 | 21.642 | 0.62 | 0.44 | 0.18 | ASTM D4253 and D4254 | Chen and Kulhawy (2014) |
| 598 | Quaternary Gravel |  |  |  |  | 16.979 | 21.789 | 0.57 | 0.36 | 0.22 | ASTM D4253 and D4254 | Chen and Kulhawy (2014) |
| 599 | Quaternary Gravel |  |  |  |  | 5.509 | 23.002 | 0.70 | 0.47 | 0.24 | ASTM D4253 and D4254 | Chen and Kulhawy (2014) |
| 600 | Quaternary Gravel |  |  |  |  | 11.548 | 25.461 | 0.60 | 0.40 | 0.20 | ASTM D4253 and D4254 | Chen and Kulhawy (2014) |
| 601 | Quaternary Gravel |  |  |  |  | 13.064 | 38.226 | 0.67 | 0.39 | 0.27 | ASTM D4253 and D4254 | Chen and Kulhawy (2014) |
| 602 | Quaternary Gravel |  |  |  |  | 11.371 | 39.542 | 0.54 | 0.30 | 0.25 | ASTM D4253 and D4254 | Chen and Kulhawy (2014) |
| 603 | Quaternary Gravel |  |  |  |  | 12.002 | 42.027 | 0.66 | 0.42 | 0.24 | ASTM D4253 and D4254 | Chen and Kulhawy (2014) |
| 604 | Quaternary Gravel |  |  |  |  | 11.910 | 43.770 | 0.60 | 0.38 | 0.21 | ASTM D4253 and D4254 | Chen and Kulhawy (2014) |
| 605 | Quaternary Gravel |  |  |  |  | 14.111 | 50.459 | 0.69 | 0.41 | 0.27 | ASTM D4253 and D4254 | Chen and Kulhawy (2014) |
| 606 | Quaternary Gravel |  |  |  |  | 9.450 | 56.616 | 0.68 | 0.40 | 0.27 | ASTM D4253 and D4254 | Chen and Kulhawy (2014) |
| 607 | Quaternary Gravel |  |  |  |  | 5.725 | 60.174 | 0.45 | 0.23 | 0.22 | ASTM D4253 and D4254 | Chen and Kulhawy (2014) |
| 608 | Quaternary Gravel |  |  |  |  | 14.553 | 63.956 | 0.43 | 0.22 | 0.21 | ASTM D4253 and D4254 | Chen and Kulhawy (2014) |
| 609 | Quaternary Gravel |  |  |  |  | 19.507 | 67.976 | 0.41 | 0.20 | 0.21 | ASTM D4253 and D4254 | Chen and Kulhawy (2014) |
| 610 | Quaternary Gravel |  |  |  |  | 8.749 | 70.317 | 0.44 | 0.23 | 0.21 | ASTM D4253 and D4254 | Chen and Kulhawy (2014) |
| 611 | Quaternary Gravel |  |  |  |  | 5.595 | 121.701 | 0.45 | 0.26 | 0.19 | ASTM D4253 and D4254 | Chen and Kulhawy (2014) |
| 612 | Gravel Fill |  |  |  |  | 2.085 | 11.896 | 0.86 | 0.53 | 0.33 | ASTM D4253 and D4254 | Chen and Kulhawy (2014) |
| 613 | Gravel Fill |  |  |  |  | 2.509 | 25.901 | 0.75 | 0.40 | 0.35 | ASTM D4253 and D4254 | Chen and Kulhawy (2014) |
| 614 | Gravel Fill |  |  |  |  | 2.710 | 29.058 | 0.78 | 0.46 | 0.32 | ASTM D4253 and D4254 | Chen and Kulhawy (2014) |
| 615 | Volcanic Gravel |  |  |  |  | 3.985 | 136.817 | 0.41 | 0.21 | 0.20 | ASTM D4253 and D4254 | Chen and Kulhawy (2014) |
| 616 | Volcanic Gravel |  |  |  |  | 5.140 | 186.768 | 0.41 | 0.21 | 0.20 | ASTM D4253 and D4254 | Chen and Kulhawy (2014) |
| 617 | Volcanic Gravel |  |  |  |  | 5.100 | 199.841 | 0.41 | 0.21 | 0.20 | ASTM D4253 and D4254 | Chen and Kulhawy (2014) |
| 618 | Volcanic Gravel |  |  |  |  | 16.211 | 261.949 | 0.41 | 0.21 | 0.20 | ASTM D4253 and D4254 | Chen and Kulhawy (2014) |
| 619 | Volcanic Gravel |  |  |  |  | 9.093 | 325.269 | 0.41 | 0.21 | 0.20 | ASTM D4253 and D4254 | Chen and Kulhawy (2014) |
| 620 | Clean Ahmedabad Sand |  |  |  |  | 0.375 |  | 0.68 | 0.42 | 0.26 | Indian Standard IS: 2720 part 3-1980 and IS: 2720 part 14-1983 | Zuo and Baudet (2015), Dash et al. (2010) |
| 621 | Sand from Stava Tailings |  |  |  |  | 0.210 |  | 1.07 | 0.62 | 0.45 | ASTM D4253 and D4254 | Zuo and Baudet (2015), Carrera et al (2011) |
| ***Data #*** | ***Soil Type*** | ***Soil Classification*** | ***R*** | ***S*** | ***FC*** | ***D_50_ (mm)*** | ***Cu*** | ***e_max_*** | ***e_min_*** | ***e_max_-e_min_*** | ***Test Methodology*** | ***Reference*** |
| 622 | F55 Foundry Sand |  |  |  |  | 0.250 |  | 0.80 | 0.61 | 0.19 | ASTM D4254 | Zuo and Baudet (2015), Thevanayagam et al. (2002) |
| 623 | Hokksund Sand |  |  |  |  | 0.440 |  | 0.95 | 0.57 | 0.38 | NA | Zuo and Baudet (2015), Yang et al.(2006) |
| 624 | Monterey no.0/30 Sand |  |  |  |  | 0.430 |  | 0.82 | 0.63 | 0.19 | (ASTM D 4253) and D4254, Method B and C | Zuo and Baudet (2015), Polito and Martin(2001) |
| 625 | Clean Sand M31 from Assyros in Greece |  |  |  |  | 0.300 |  | 0.84 | 0.58 | 0.26 | ASTM D4253 and D4254 | Zuo and Baudet (2015), Papadopoulou andTika (2008) |
| 626 | Sxinias-Marathon Sand |  |  |  |  | 0.120 |  | 1.04 | 0.66 | 0.38 | ASTM D4254 and ASTM D4253 | Zuo and Baudet (2015), Xenaki and Athanasopoulos (2003) |
| 627 | Leighton Buzzard Sand |  |  |  |  | 0.900 |  | 0.79 | 0.52 | 0.27 | NA | Zuo and Baudet (2015), Cabalar (2010) |
| 628 | Non-plastic quarry dust around Bangalore |  |  |  |  | 0.037 |  | 1.63 | 0.65 | 0.98 | Indian Standard IS: 2720 part 3-1980 and IS: 2720 part 14-1983 | Zuo and Baudet (2015), Dash etal.(2010) |
| 629 | Non-plastic Crushed silica fines |  |  |  |  | 0.010 |  | 2.10 | 0.63 | 1.47 | ASTM D4254 | Zuo and Baudet (2015), Thevanayagam et al. (2002) |
| 630 | Chengbeinon-plastic silt |  |  |  |  | 0.032 |  | 1.41 | 0.73 | 0.68 | NA | Zuo and Baudet (2015), Yang etal.(2006) |
| 631 | Fine-grained portion of Yatesville Silty Sand |  |  |  |  | 0.030 |  | 1.72 | 0.73 | 1.00 | (ASTM D 4253) and D4254, Method B and C | Zuo and Baudet (2015), Polito andMartin(2001) |
| 632 | Non-plastic silt from Assyrosin |  |  |  |  | 0.020 |  | 1.66 | 0.66 | 1.01 | ASTM D4253 and D4254 | Zuo and Baudet (2015), Papadopoulou andTika (2008) |
| 633 | Sxinias-Marathon fines |  |  |  |  | 0.020 |  | 1.71 | 0.66 | 1.05 | ASTM D4254 and ASTM D4253 | Zuo and Baudet (2015), Xenaki and Athanasopoulos (2003) |
| 634 | Mica |  |  |  |  | 0.130 |  | 3.00 | 2.20 | 0.80 | NA | Zuo and Baudet (2015), Cabalar (2010) |
| 635 | Upper Peninsula, Michigan |  | 0.51 | 0.69 |  | 0.600 | 2.800 | 0.85 | 0.54 | 0.31 | NA | Zheng and Hryciw (2016) |
| 636 | Kızılırmak Sand | SP |  |  | 3.7 | 1.132 | 8.200 | 0.80 | 0.45 | 0.35 | Non-Standard Procedures | Personel Communication |

**References**

[1] Arulmoli, K., Muraleetharan, K.K., Hosain, M.M. and Fruth., L.S. VELACS Laboratory Testing Program, Soil Data Report. The Earth Technology Corporation, Irvine, California, Report to the National Science Foundation, Washington D.C. (1992)

[2] Baldi, G., Bellotti, R., Ghionna, V., Jamiolkowski, M., and Pasqualini, E. Cone Resistance of a Dry Medium Sand. Proc. 10th Intl. Conf. Soil Mach. & Fndn. Eng. (2), Stockholm, 427-432 (1981)

[3] Baldi, G., Bellotti, R., Ghionna, V., Jamiolkowski, M., and Pasqualini, E. Interpretation of CPTs and CPTUs: Drained Penetration of Sands, Proc. 4th Intl. Geot. Sam. on Field Inst. & In-Situ Meas., Nanyang Tech. Inst., Singapore, 143-156 (1986)

[4] Barden, L., Madedor, A. O., & Sides, G. R. Volume change characteristics of unsaturated clay. Journal of Soil Mechanics & Foundations Div. (1969)

[5] Bareither, C. A., Edil, T. B., Benson, C. H., & Mickelson, D. M. Geological and physical factors affecting the friction angle of compacted sands. Journal of geotechnical and geoenvironmental engineering, 134(10), 1476-1489 (2008)

[6] Baxter, C. D., & Mitchell, J. K. Experimental study on the aging of sands. Journal of Geotechnical and Geoenvironmental Engineering, 130(10), 1051-1062 (2004)

[7] Been, K., & Jefferies, M. G. A state parameter for sands. Géotechnique, 35(2), 99-112 (1985)

[8] Been, K., Lingnau, B. E., Crooks, J. H. A., & Leach, B. Cone penetration test calibration for Erksak (Beaufort Sea) sand. Canadian Geotechnical Journal, 24(4), 601-610 (1987).

[9] Bellotti, R., Bizzi, G., Ghionna, V., Jamiolkowski, M., Marchetti, S., and Pasqualini, E. Preliminary Calibration Tests of Electrical Cone and Flat Dilatometer in Sand, Proc. 8th Eur. Conf. Soil Mech. & Fndn. Eng. (2), Brighton, 195-200 (1979)

[10] Bishop, A. W., & Green, G. E. The influence of end restraint on the compression strength of a cohesionless soil. Geotechnique, 15(3), 243-266 (1965).

[11] Bobei, D. C., Lo, S. R., Wanatowski, D., Gnanendran, C. T., & Rahman, M. M. Modified state parameter for characterizing static liquefaction of sand with fines. Canadian Geotechnical Journal, 46(3), 281-295 (2009).

[12] Bolton, M. D. Strength and dilatancy of sands. Geotechnique, 36(1), 65-78 (1986)

[13] Bolton, M. D. The strength and dilatancy of sands. Géotechnique, 37(2) (1987)

[14] Borden, R. H. Boundary displacement induced by DMT penetration. In Proceedings of the First International Symposium on Calibration Chamber Testing/ISOCCT1, An-Bin Huang, Ed., Potsdam, New York (pp. 101-118) (1991)

[15] Brandon, T.L., Clough, G.W. and Rajardjo, R.P. Fabrication of silty sand specimens for large and small scale tests. Geotechnical Testing Journal, 14, 1, 46–55 (1991).

[16] Cabalar, A. F. Applications of the oedometer, triaxial and resonant column tests to the study of micaceous sands. Engineering Geology, 112(1-4), 21-28 (2010).

[17] Cabalar, A. F., Dulundu, K., & Tuncay, K. Strength of various sands in triaxial and cyclic direct shear tests. Engineering Geology, 156, 92-102 (2013).

[18] Canou, J., El Hachem, M., Kattan, A., & Juran, I. Mini piezo-cone (M-CPTU) investigation related to sand liquefaction analysis. In Proceeding of International Symposium of Penetration Testing I. (1988).

[19] Carrera, A., Coop, M., Lancellotta, R. Influence of grading on the mechanical behaviour of Stava tailings. Géotechnique 61 (11), 935–946 (2011)

[20] Castro, G. Liquefaction of sands. Ph. D. Thesis, Harvard Soil Mech. (1969).

[21] Cetin and Cakir (2019). Personal communication.

[22] Chapman, G. A., & Donald, I. B. Interpretation of static penetration tests in sand. In International Conference on Soil Mechanics and Foundation Engineering, 10th, 1981, Stockholm, Sweden (Vol. 2) (1981).

[23] Chen, J. R., & Kulhawy, F. H. Characteristics and intercorrelations of index properties for cohesionless gravelly soils. In Geo-Congress 2014: Geo-characterization and Modeling for Sustainability (pp. 1-13) (2014).

[24] Cho, G. C., Dodds, J., & Santamarina, J. C. Particle shape effects on packing density, stiffness, and strength: natural and crushed sands. Journal of geotechnical and geoenvironmental engineering, 132(5), 591-602 (2006)

[25] Chong, M.K. Density Changes of Sand on Cone Penetration Resistances, Proc. 1st Intl. Symp. Pen. Test. (2), Orlando, 707-714 (1988)

[26] Chung, R. M., Yokel, F. Y., & Drnevich, V. P. Evaluation of dynamic properties of sands by resonant column testing. Geotechnical Testing Journal, 7(2), 60-69 (1984)

[27] Coop, M., and Lee, I. K. The behaviour of granular soils at elevated stresses. Predictive Soil Mechanics, Proc., Wroth Memorial Symp., Thomas Telford, London, 186–198 (1993)

[28] Cornforth, D. H. Some experiments on the influence of strain conditions on the strength of sand. Geotechnique, 14(2), 143-167 (1964)

[29] Cornforth, D. H. Prediction of drained strength of sands from relative density measurements. In Evaluation of relative density and its role in geotechnical projects involving cohesionless soils. ASTM International (1973)

[30] Cubrinovski, M., & Ishihara, K. Modelling of sand behaviour based on state concept. Soils and Foundations, 38(3), 115-127 (1998)

[31] Cubrinovski, M., & Ishihara, K. Maximum and minimum void ratio characteristics of sands. Soils and foundations, 42(6), 65-78 (2002)

[32] Dash, H. K., Sitharam, T. G., & Baudet, B. A. Influence of non-plastic fines on the response of a silty sand to cyclic loading. Soils and Foundations, 50(5), 695-704 (2010)

[33] De Beer, E. E. The scale effect in the transposition of the results of deep-sounding tests on the ultimate bearing capacity of piles and caisson foundations. Geotechnique, 13(1) 39-75 (1963)

[34] DeJong, J. T., & Christoph, G. G. Influence of particle properties and initial specimen state on one-dimensional compression and hydraulic conductivity. Journal of geotechnical and geoenvironmental engineering, 135(3), 449-454 (2009)

[35] Ezaoui, A., & Benedetto, H. D. Experimental measurements of the global anisotropic elastic behaviour of dry Hostun sand during triaxial tests, and effect of sample preparation. Géotechnique, 59(7), 621-635 (2009)

[36] Fourie, A. B., & Papageorgiou, G. Defining an appropriate steady state line for Merriespruit gold tailings. Canadian Geotechnical Journal, 38(4), 695-706 (2001)

[37] Georgiannou, V. N., & Konstadinou, M. Torsional shear behavior of anisotropically consolidated sands. Journal of Geotechnical and Geoenvironmental Engineering, 140(2), 04013017 (2013)

[38] Goto, S., Park, C., Tatsuoka, F., & Molenkamp, F. Quality of the lubrication layer used in element tests on granular materials. Soils and Foundations, 33(2), 47-59 (1993)

[39] Greeuw, G., Smits, F. P., & Van Driel, P. Cone penetration tests in dry Oosterschelde sand and the relation with a cavity expansion model. In Proceedings, 1st International Symposium on Penetration Testing, ISOPT-1, Balkema, Rotterdam, the Netherlands (Vol. 2, pp. 771-776) (1988)

[40] Guo, P., and Su, X. Shear strength, interparticle locking, and dilatancy of granular materials. Can. Geotech. J., 44(5), 579–591 (2007)

[41] Gutierrez, M., Ishihara, K., & Towhata, I. Flow theory for sand during rotation of principal stress direction. Soils and foundations, 31(4), 121-132 (1991)

[42] Herle, I., & Gudehus, G. Determination of parameters of a hypoplastic constitutive model from properties of grain assemblies. Mechanics of Cohesive‐frictional Materials: An International Journal on Experiments, Modelling and Computation of Materials and Structures, 4(5), 461-486 (1999)

[43] Hettler, A. Verschiebungen starrer und elastischer Gründungskörper in Sand bei monotoner und zyklischer Belastung (Vol.90). Institut für Bodenmechanik und Felsmechanik der Universität Fridericiana (1981)

[44] Hird, C. C., & Hassona, F. A. K. Some factors affecting the liquefaction and flow of saturated sands in laboratory tests. Engineering Geology, 28(1-2), 149-170 (1990)

[45] Hirschfeld, R. C., & Poulos, S. J. High-pressure triaxial tests on a compacted sand and an undisturbed silt. In Laboratory shear testing of soils. ASTM International (1964)

[46] Holden, J. C. The determination of deformation and shear strength parameters for sands using the electrical friction-cone penetrometer (No. Publication No. 110 Monograph) (1976)

[47] Hoque, E., & Tatsuoka, F. Anisotropy in elastic deformation of granular materials. Soils and foundations, 38(1), 163-179 (1998)

[48] Houlsby, G. T., & Hitchman, R. Calibration chamber tests of a cone penetrometer in sand. Geotechnique, 38(1), 39-44 (1988)

[49] Huang, A. B. Calibration chamber testing: proceedings of the First International Symposium on Calibration Chamber Testing/ISOCCT1, Potsdam, New York, 28-29 June 1991. Elsevier. (1991)

[50] Huntsman, S.R., Mitchell, J.K., KleJbuk, L.W. and Shinde, S.B. Lateral Stress Measurement During Cone Penetration, Use of In-Situ Tests in Geot. Eng. (GSP 6), Ed. S.P. Clemence, ASCE, New York, 617-634 (1986)

[51] Hyodo, M., Murata, H., Yasufuku, N., & Fujii, T. Undrained cyclic shear strength and residual shear strain of saturated sand by cyclic triaxial tests. Soils and Foundations, 31(3), 60-76 (1991)

[52] Ishihara, K., Silver, M. L., & Kitagawa, H. Cyclic strengths of undisturbed sands obtained by large diameter sampling. Soils and Foundations, 18(4), 61-76 (1978)

[53] Ishihara, K., Silver, M. L., & Kitagawa, H. Cyclic strength of undisturbed sands obtained by a piston sampler. Soils and Foundations, 19(3), 61-76 (1979)

[54] Ishihara, K., & Koga, Y. Case studies of liquefaction in the 1964 Niigata earthquake. Soils and foundations, 21(3), 35-52 (1981)

[55] Jefferies, M. G. Been K. Soil Liquefaction: a critical state approach (2006)

[56] Kokusho, T., & Yoshida, Y. SPT N-value and S-wave velocity for gravelly soils with different grain size distribution. Soils and Foundations, 37(4), 105-113 (1997)

[57] Kumar, J., & Madhusudhan, B. N. Dynamic properties of sand from dry to fully saturated states. Geotechnique, 62(1), 45-54 (2012)

[58] Ladanyi, B. Etude des relations entre les contraintes et les deformations lors du cisaillement des sols pulverulents. Verlag nicht ermittelbar (1960)

[59] Lade, P. V., & Duncan, J. M. Cubical triaxial tests on cohesionless soil. Journal of Soil Mechanics & Foundations Div, 99 (Proc Paper 10057) (1973)

[60] Lade, Poul V., and Jerry A. Yamamuro. "Effects of nonplastic fines on static liquefaction of sands." Canadian Geotechnical Journal 34.6 (1997): 918-928.

[61] Lade, P. V., Liggio, C. D., & Yamamuro, J. A. Effects of non-plastic fines on minimum and maximum void ratios of sand. Geotechnical testing journal, 21, 336-347 (1998)

[62] Lambrechts, J. R., & Leonards, G. A. Effects of stress history on deformation of sand. Journal of Geotechnical and Geoenvironmental Engineering, 104(ASCE 14170 Proc Paper) (1978)

[63] Lee, K. L., & Seed, H. B. Drained strength characteristics of sands. Journal of Soil Mechanics & Foundations Div. (1967)

[64] Lings, M. L., & Dietz, M. S. An improved direct shear apparatus for sand. Geotechnique, 54(4), 245-256 (2004)

[65] Lo Presti, D. Mechanical behaviour of Ticino sand from resonant column tests (Doctoral dissertation, Ph. D. thesis, Politecnico di Torino, Torino, Italy) (1987)

[66] Lo Presti, D., Pedroni, S., & Crippa, V. Maximum dry density of cohesionless soils by pluviation and by ASTM D 4253-83: A comparative study. Geotechnical Testing Journal, 15(2), 180-189 (1992)

[67] Lunne, T., Christoffersen, H. P., & Tjelta, T. I. Engineering of piezocono results in North Sea Clays. Proc. XI ICMSMFE, San Francisco (1985)

[68] Lunne, T. Practical use of CPT correlations in sand based on calibration chamber tests. In Proc. of the first International Symposium on Calibration Chamber testing (pp. 225-236) (1991)

[69] Marachi, N. D., Chan, C. K., Bolton Seed, H., & Duncan, J. M. Strength and deformation of Rockfill. Report NTE-69-5 Dept. of Civil Engineering/Geotechnical Engineering-Univ. of California-Berkeley. (140p) (1969)

[70] Mayne, P. W., and Kulhawy, F. H. Calibration chamber database and boundary effects correction for CPT data. Proc., 1st Int. Symp. on Calibration Chamber Testing (ISOCCTI). Elsevier, Potsdam, N.Y., 257-264 (1991)

[71] Miura, S., & Kawamura, S. A procedure minimizing membrane penetration effects in undrained triaxial test. Soils and foundations, 36(4), 119-126 (1996)

[72] Moroto, N., and Ishii, T. Shear strength of uni-sized gravels under triaxial compression. Soils Found., 30(2), 23–32 (1990)

[73] Papadopoulou, A., & Tika, T. The effect of fines on critical state and liquefaction resistance characteristics of non-plastic silty sands. Soils and foundations, 48(5), 713-725 (2008)

[74] Parkin, A.K., Holden, J., Aamot, K., Last, N. and Lunne, T. Laboratory Investigation of CPTs in Sand, Rpt. 52108-9, Norwegian Geot. Inst., Oslo, 45 p. (1980)

[75] Polito, C. P., & Martin II, J. R. Effects of nonplastic fines on the liquefaction resistance of sands. Journal of Geotechnical and Geoenvironmental Engineering, 127(5), 408-415 (2001)

[76] Pitman, T. D., Robertson, P. K., & Sego, D. C. Influence of fines on the collapse of loose sands. Canadian Geotechnical Journal, 31(5), 728-739 (1994)

[77] Rix, G. J., & Stokoe, K. H. Correlation of initial tangent modulus and cone penetration resistance. Calibration chamber testing, 351-361 (1991)

[78] Roberts, J. E. Sand compression as a factor in oil field subsidence. Ph.D. thesis, Massachusetts Institute of Technology, Cambridge, MA. (1964)

[79] Robertson, P.K., Wride, C.E., List, B.R., Atukorala, U., Biggar, K.W., Byrne, P.M., Campanella, R.G., Cathro, D.C., Chan, D.H., Czajewski, K., Finn, W.D.L., Gu, W.H., Hammamji, Y., Hofmann, B.A., Howie, J.A., Hughes, J., Imrie, A.S., Konrad, J.-M., Küpper, A., Law, K.T., Lord, E.R.F., Monahan, P.A., Morgenstern, N.R., Phillips, R., Piché, R., Plewes, H.D., Scott, D., Sego, D.C., Sobkowicz, J., Stewart, R.A, Watts, B.D, Woeller, D.J., Youd, T.L. and Zavodni, Z. The CANLEX project: summary and conclusions. Canadian Geotechnical Journal, 37, 3, 563–591 (2000)

[80] Rousé, P. C., Fannin, R. J., & Shuttle, D. A. Influence of roundness on the void ratio and strength of uniform sand. Géotechnique, 58(3), 227-231 (2008)

[81] Rowe, P. W., & Barden, L. Importance of free ends in triaxial testing. Journal of Soil Mechanics & Foundations Div, 90(Proc. Paper 3753) (1964)

[82] Rowe, P. W. The relation between the shear strength of sands in triaxial compression, plane strain and direct. Geotechnique, 19(1), 75-86 (1969)

[83] Sakai, T., & Tanaka, T. Scale effect of a shallow circular anchor in dense sand. Soils and Foundations, 38(2), 93-99 (1998)

[84] Salgado, R., Bandini, P., & Karim, A. Shear strength and stiffness of silty sand. Journal of Geotechnical and Geoenvironmental Engineering, 126(5), 451-462 (2000)

[85] Santamarina, J. C., & Cho, G. C. Determination of critical state parameters in sandy soils—simple procedure. Geotechnical testing journal, 24(2), 185-192 (2001)

[86] Schmertmann, J. H. Guidelines for cone penetration test: performance and design (No. FHWA-TS-78-209). United States. Federal Highway Administration (1978)

[87] Seed, H.B., Seed, R.B., Harder, L.F., and Jong, H.-L. Re-evaluation of the slide in the lower San Fernando dam in the earthquake of February 9, 1971. Report UCB/EERC-88/04, Earthquake Engineering Research Centre, University of California at Berkeley (1988)

[88] Shahnazari, H., & Rezvani, R. Effective parameters for the particle breakage of calcareous sands: An experimental study. Engineering geology, 159, 98-105 (2013)

[89] Shamoto, Y., Sato, M., & Zhang, J. Simplified estimation of earthquake-induced settlements in saturated sand deposits. Soils and Foundations, 36(1), 39-50 (1996)

[90] Skempton, A. W. Standard penetration test procedures and the effects in sands of overburden pressure, relative density, particle size, ageing and overconsolidation. Geotechnique, 36(3), 425-447 (1986)

[91] Sladen, J. A., D'hollander, R. D., & Krahn, J. The liquefaction of sands, a collapse surface approach. Canadian geotechnical journal, 22(4), 564-578 (1985)

[92] Stroud, M. A. The behaviour of sand at low stress levels in the simple-shear apparatus (Doctoral dissertation, University of Cambridge. (1971)

[93] Sukumaran, B., & Ashmawy, A. K. Quantitative characterisation of the geometry of discret particles. Geotechnique, 51(7), 619-627 (2001)

[94] Sweeney, B.P. Liquefaction Evaluation Using a Miniature Cone Penetrometer and Large Scale Calibration Chamber, PhD Thesis, Stanford Univ., 281 p. (1987)

[95] Thevanayagam, S. Effect of fines and confining stress on undrained shear strength of silty sands. Journal of Geotechnical and Geoenvironmental Engineering, 124(6), 479-491 (1998)

[96] Thevanayagam, S., Shenthan, T., Mohan, S., & Liang, J. Undrained fragility of clean sands, silty sands, and sandy silts. Journal of geotechnical and geoenvironmental engineering, 128(10), 849-859 (2002)

[97] Thomann, T. G. Stiffness and strength changes in cohensionless soils due to stress history and dynamic disturbance. Ph.D. thesis, Univ. of Michigan, Ann Arbor, MI. (1990)

[98] Thomas, D. Deep Sounding Test Results and the Settlement of Spread Footings on Normally Consolidated Sands, Geotechnique, 20 (4), 472-488 (1968)

[99] Tsomokos, A., & Georgiannou, V. N. Effect of grain shape and angularity on the undrained response of fine sands. Canadian Geotechnical Journal, 47(5), 539-551 (2010)

[100] Veismanis, A. Laboratory investigation of electrical friction cone penetrometers in sands. In Proceedings of European Symposium on Penetration Testing, Stockholm (Vol. 2, pp. 407-420) (1974)

[101] Vesic, A. S., & Clough, G. W. Behavior of granular materials under high stresses. Journal of Soil Mechanics & Foundations Div. (1968)

[102] Villet, W. C., & Mitchell, J. K. Cone resistance, relative density and friction angle. In Cone penetration testing and experience. ASCE. (pp. 178-208). (1981)

[103] Xenaki, V.C., Athanasopoulos, G.A. Liquefaction resistance of sand–silt mixtures: an experimental investigation of the effect of fines. Soil Dyn.Earthquake Eng. 23 (3). 1–12 (2003)

[104] Yamashita, S., & Toki, S. Effects of fabric anisotropy of sand on cyclic undrained triaxial and torsional strengths. Soils and foundations. 33(3), 92-104 (1993)

[105] Yang, S. L., Sandven, R., & Grande, L. Steady-state lines of sand–silt mixtures. Canadian Geotechnical Journal. 43(11), 1213-1219 (2006)

[106] Yang, Z. X., Jardine, R. J., Zhu, B. T., Foray, P., & Tsuha, C. D. H. C. Sand grain crushing and interface shearing during displacement pile installation in sand. Géotechnique. 60(6), 469 (2010)

[107] Yasin, S. J. M., & Safiullah, A. M. M. Effect of particle characteristics on the strength and volume change behaviour of sand. J. Civ. Eng. 31(2), 127-148 (2003)

[108] Youd, T. L. Factors controlling maximum and minimum densities of sands. In Evaluation of relative density and its role in geotechnical projects involving cohesionless soils. ASTM International. (1973)

[109] Zelasko, J. S., Krizek, R. J., & Edil, T. B. Shear behavior of sands as a function of grain characteristics. 1, 55-64 (1975)

[110] Zen, K., & Yamazaki, H. Mechanism of wave-induced liquefaction and densification in seabed. Soils and foundations. 30(4), 90-104 (1990)

[111] Zhang, J. M., Shamoto, Y. A. S. U. H. I. R. O., & Tokimatsu, K. Moving critical and phase-transformation stress state lines of saturated sand during undrained cyclic shear. Soils and foundations. 37(2), 51-59 (1997)

[112] Zheng, J., & Hryciw, R. D. Roundness and sphericity of soil particles in assemblies by computational geometry. Journal of Computing in Civil Engineering. 30(6), 04016021 (2016)

[113] Zlatovic, S. (1994). Residual strength of silty soils (Doctoral dissertation, 東京大学).

[114] Zuo, L., & Baudet, B. A. Determination of the transitional fines content of sand-non plastic fines mixtures. Soils and Foundations. 55(1), 213-219 (2015)
